# Supplementary material for: How the Eukaryotic Replisome Achieves Rapid and Efficient DNA Replication
Source: Mol Cell. 2017 Jan 5;65(1):105–16. doi: 10.1016/j.molcel.2016.11.017 (PMC5222725; doi:10.1016/j.molcel.2016.11.017)
Supplement: Document S1. Supplemental Experimental Procedures, Figures S1–S7, and Tables S1–S4 [file mmc1.pdf]

Molecular Cell, Volume *65*

Supplemental Information

How the Eukaryotic Replisome Achieves Rapid and Efficient DNA Replication

Joseph T.P. Yeeles, Agnieska Janska, Anne Early, and John F.X. Diffley

Figure S1

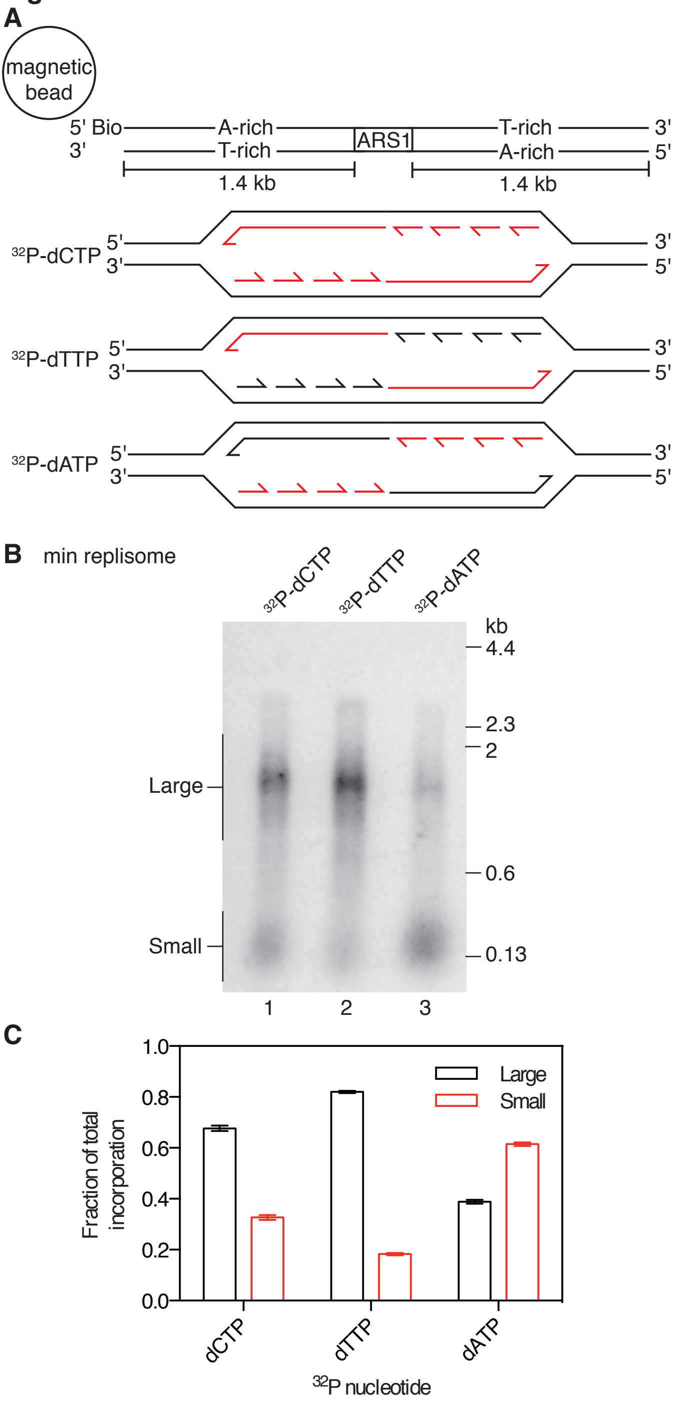

A
magnetic bead
5' Bio
A-rich
T-rich
ARS1
3'
1.4 kb
1.4 kb
5'
^{32}P-dCTP
^{32}P-dTTP
^{32}P-dATP
B
min replisome
kb
4.4
2.3
2
0.6
0.13
Large
Small
1
2
3
C
Fraction of total incorporation
1.0
0.8
0.6
0.4
0.2
0.0
Large
Small
dCTP
dTTP
dATP
^{32}P nucleotide

Figure S2

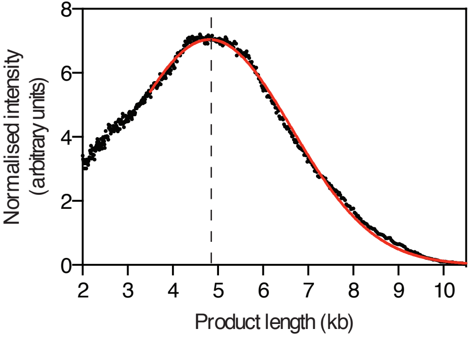

8
6
4
2
0
Normalised intensity
(arbitrary units)
2
3
4
5
6
7
8
9
10
Product length (kb)

Figure S3

min replisome + RFC, PCNA, Csm3/Tof1, Mrc1, Topo I

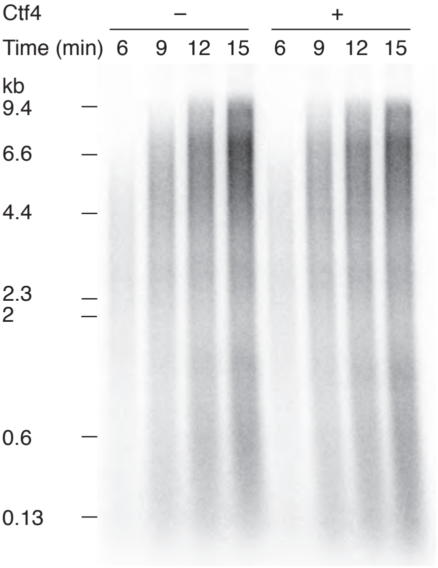

Figure S4

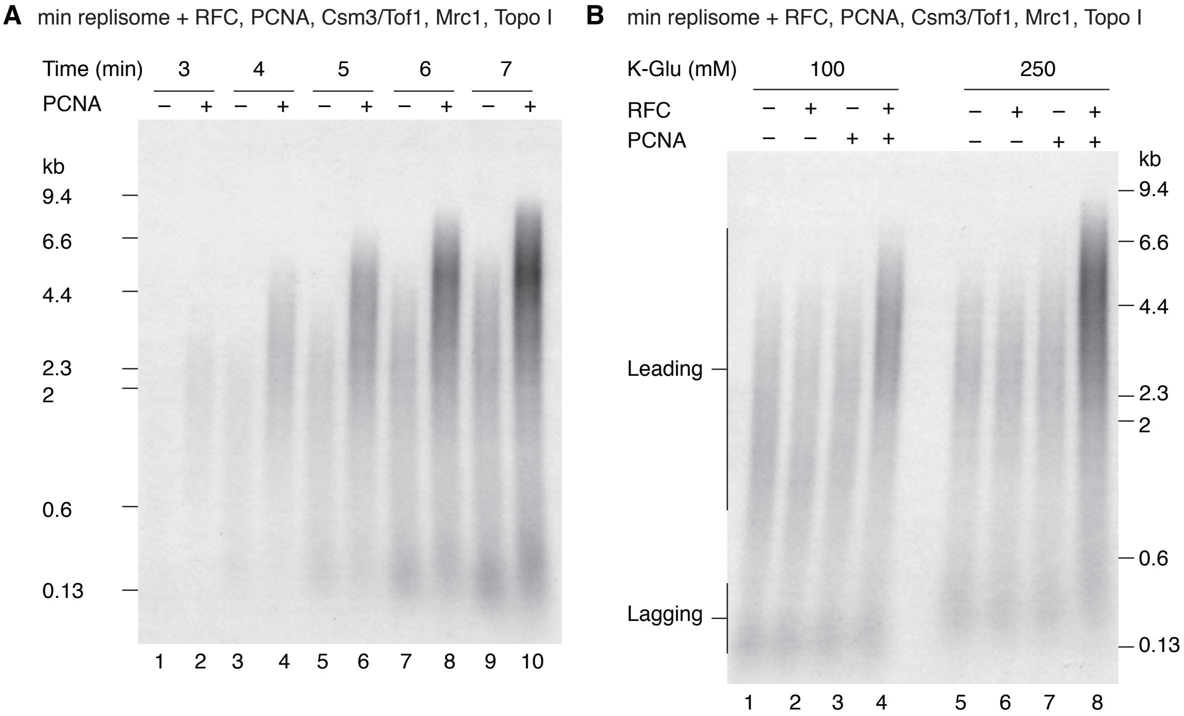

A
min replisome + RFC, PCNA, Csm3/Tof1, Mrc1, Topo I
Time (min) 3 4 5 6 7
PCNA – + – + – + – + – +
kb
9.4
6.6
4.4
2.3
2
0.6
0.13
1 2 3 4 5 6 7 8 9 10
B
min replisome + RFC, PCNA, Csm3/Tof1, Mrc1, Topo I
K-Glu (mM) 100 250
RFC – + – + – + – +
PCNA – – + + – – + +
kb
9.4
6.6
4.4
2.3
2
0.6
0.13
Leading
Lagging
1 2 3 4 5 6 7 8

Figure S5

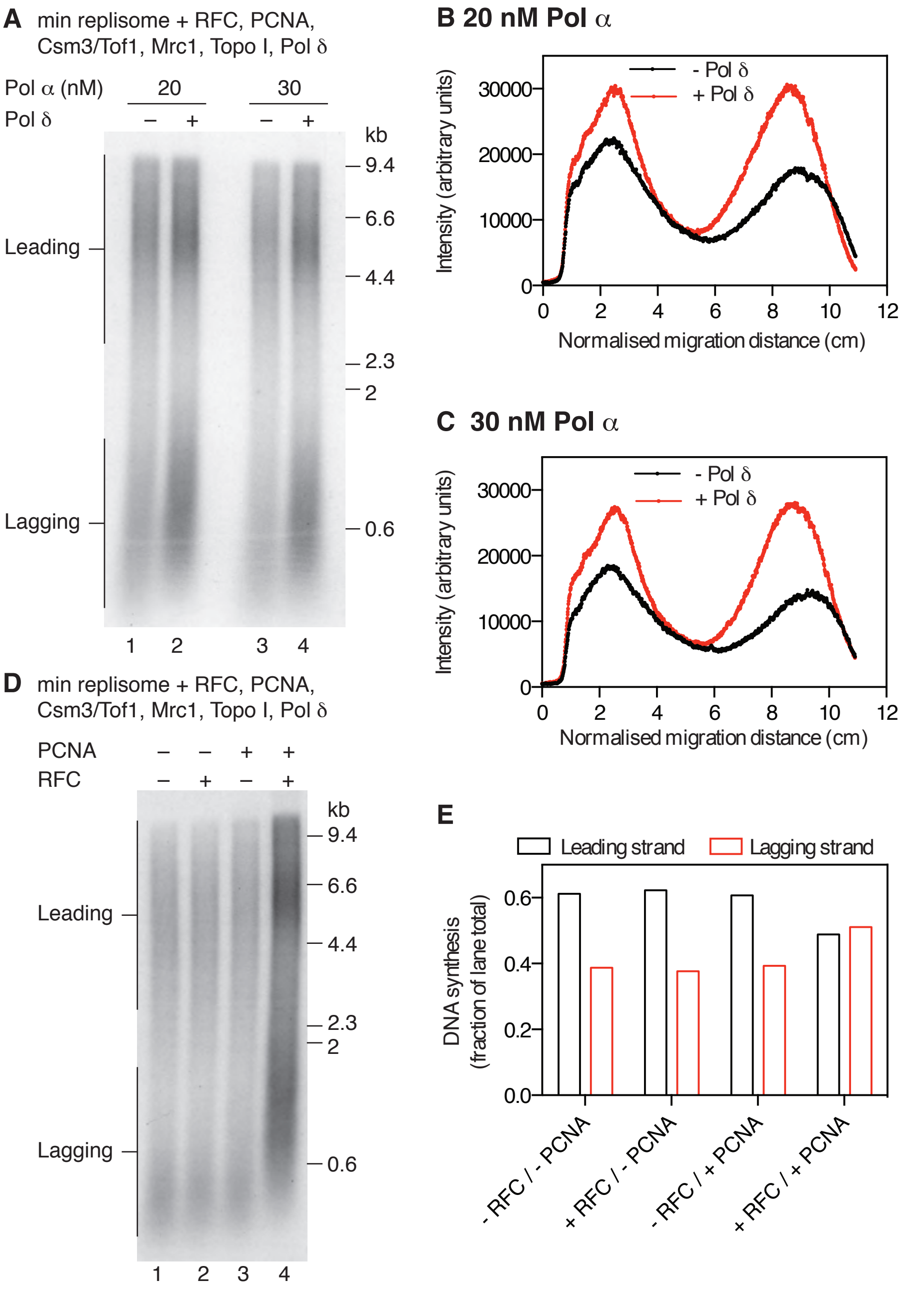
A
min replisome + RFC, PCNA, Csm3/Tof1, Mrc1, Topo I, Pol δ
Pol α (nM)
20
30
Pol δ
– + – +
kb
9.4
6.6
4.4
2.3
2
0.6
Leading
Lagging
1 2 3 4
B 20 nM Pol α
- Pol δ
+ Pol δ
Intensity (arbitrary units)
Normalised migration distance (cm)
C 30 nM Pol α
- Pol δ
+ Pol δ
Intensity (arbitrary units)
Normalised migration distance (cm)
D
min replisome + RFC, PCNA, Csm3/Tof1, Mrc1, Topo I, Pol δ
PCNA – – + +
RFC – + – +
kb
9.4
6.6
4.4
2.3
2
0.6
Leading
Lagging
1 2 3 4
E
Leading strand
Lagging strand
DNA synthesis (fraction of lane total)
- RFC / - PCNA
+ RFC / - PCNA
- RFC / + PCNA
+ RFC / + PCNA

Figure S6

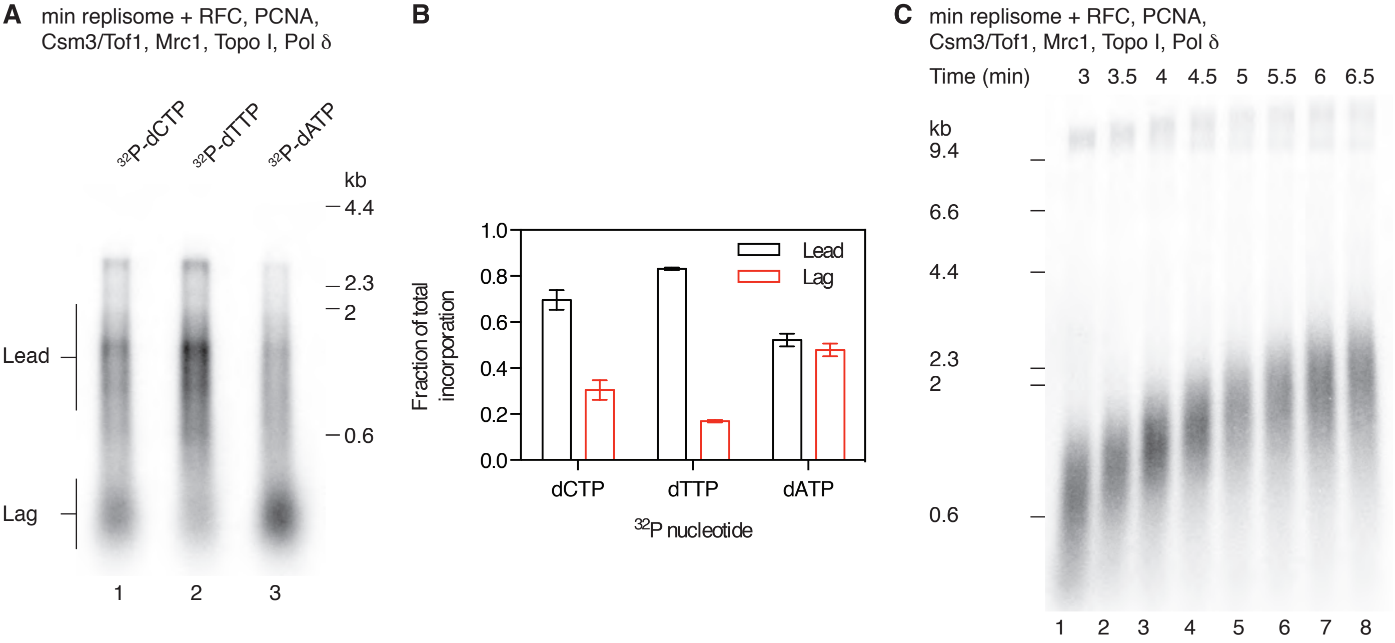
A
min replisome + RFC, PCNA, Csm3/Tof1, Mrc1, Topo I, Pol δ
32P-dCTP
32P-dTTP
32P-dATP
kb
4.4
2.3
2
0.6
Lead
Lag
1
2
3
B
Fraction of total incorporation
1.0
0.8
0.6
0.4
0.2
0.0
Lead
Lag
dCTP
dTTP
dATP
32P nucleotide
C
min replisome + RFC, PCNA, Csm3/Tof1, Mrc1, Topo I, Pol δ
Time (min) 3 3.5 4 4.5 5 5.5 6 6.5
kb
9.4
6.6
4.4
2.3
2
0.6
1 2 3 4 5 6 7 8

Figure S7

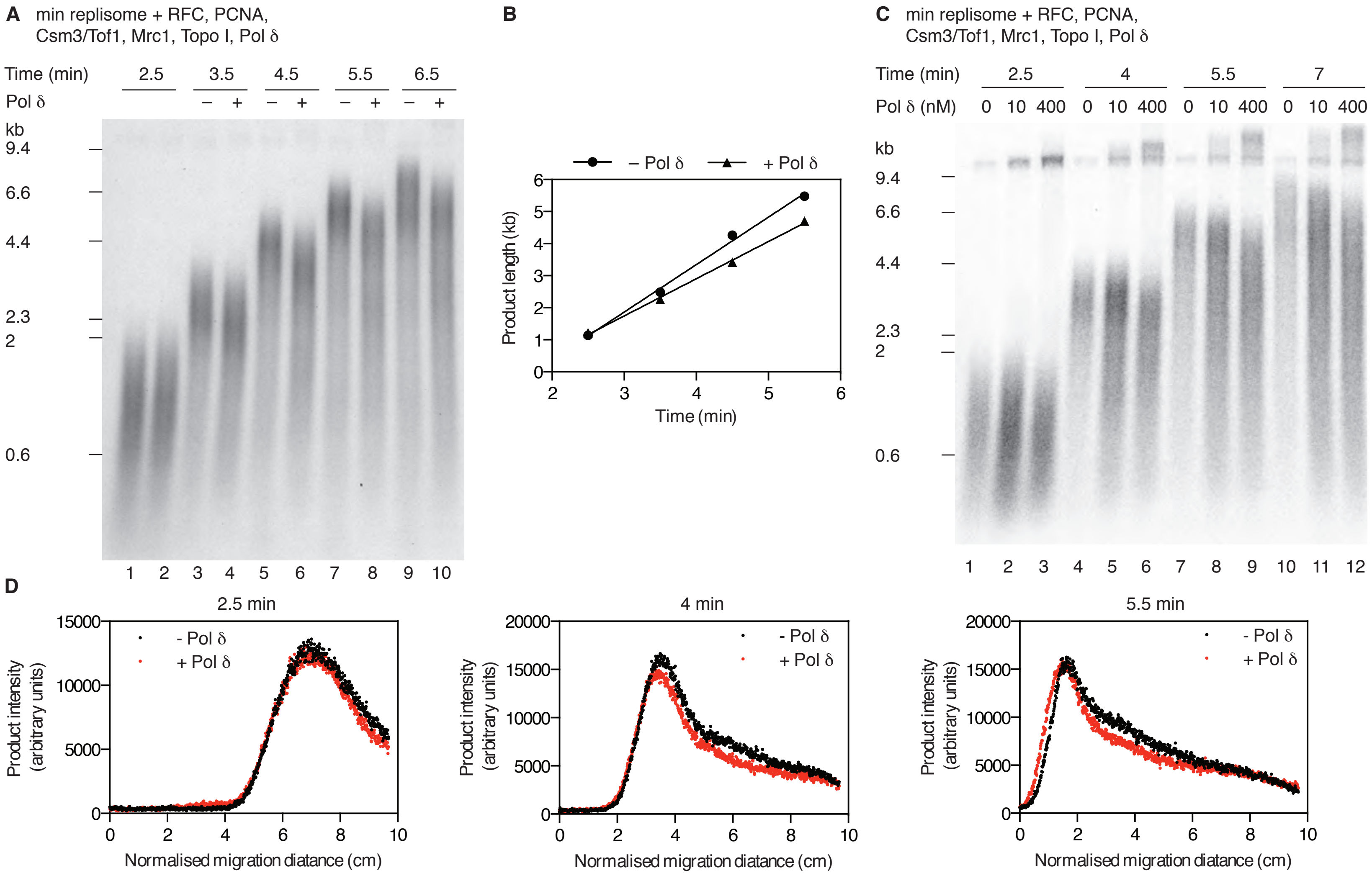
A
min replisome + RFC, PCNA, Csm3/Tof1, Mrc1, Topo I, Pol δ
Time (min) 2.5 3.5 4.5 5.5 6.5
Pol δ – + – + – + – +
kb
9.4
6.6
4.4
2.3
2
0.6
1 2 3 4 5 6 7 8 9 10
B
– Pol δ
+ Pol δ
Product length (kb)
Time (min)
C
min replisome + RFC, PCNA, Csm3/Tof1, Mrc1, Topo I, Pol δ
Time (min) 2.5 4 5.5 7
Pol δ (nM) 0 10 400 0 10 400 0 10 400 0 10 400
kb
9.4
6.6
4.4
2.3
2
0.6
1 2 3 4 5 6 7 8 9 10 11 12
D
2.5 min
4 min
5.5 min
- Pol δ
+ Pol δ
Product intensity (arbitrary units)
Normalised migration diatance (cm)

Supplemental Figure Legends

Figure S1. Related to Figure 1.

(A) Schematic representation of the template used to assign leading and lagging strand replication products. The template contains a functional 100 bp fragment of ARS1 at the centre (Liachko et al., 2013). Labelled nascent replication products are drawn in red. [α-^{32}P] dCTP will label nascent leading and lagging strands evenly. [α-^{32}P] dTTP will preferentially label nascent leading strands. Lagging strands will be preferentially labelled with [α-^{32}P] dATP.

(B) Bead-coupled replication reactions performed under origin-specific conditions. Reactions were performed for 20 min. Topo II was not present in the reaction.

(C) Quantitation of small and large products from reactions performed as described in (B). Data were normalised to the sum of small and large products for each labelled nucleotide. Data represent the average of four independent experiments and error bars show the standard error of the mean (SEM).

Figure S2. Related to Figure 1.

Normalised lane profile showing leading strand products for the 120 min time point in Figure 1B. Because products are continuously labelled in replication reactions, signal intensity is proportional to product length. To derive an unbiased distribution of replication products, signal intensity was divided by product length. Data were fit to a gaussian distribution. Products below 3.5 kb were excluded from the fit to avoided interference from the population of lagging strand products. The dotted vertical line shows the mean of the distribution.

Figure S3. Related to Figure 3.

Replication time course performed as in Figure 3A in the presence or absence of Ctf4.

Figure S4. Related to Figure 4.

(A) Time course performed as in Figure 4A but with 40 nM Pol α.

(B) Experiments performed as in Figure 4A for 6 minutes at 100 mM and 250 mM potassium glutamate. PCNA and RFC were included where indicated.

Figure S5. Related to Figure 5.

(A) Experiment conducted as in Figure 5A for 20 min.

(B and C) Lane profiles for the data in (A).

(D) Replication reaction conducted as in (A) for 15 min but with Pol δ present in all lanes.

(E) Quantitation of the leading and lagging strand products in (D).

Figure S6. Related to Figure 6.

(A) Bead coupled replication reaction performed as in Figure S1A with the following modifications. Pol ε- Δ cat was used in place of Pol ε, reactions contained 200 mM potassium glutamate and 5 nM Pol δ, and replication was performed for 30 min.

(B) Quantitation of experiments performed as described in A. Data represent the mean of 3 repeats and error bars show the SEM.

(C) Pulse chase reaction performed as in Figure 2C but with Pol ε-Δcat in place of Pol ε, and 10 nM Pol δ. The chase was added at 2 min 50 s.

Figure S7. Related to Figure 7.

(A) Pulse chase experiment performed as in Figure 6F. 400 nM Pol δ was included in the chase where indicated.

(B) Quantitation of the data in (A). Data represent the peaks of the distributions of leading strand products.

(C) Pulse chase reaction essentially as performed in (A), except that Pol δ was included from the beginning of the reactions where indicated.

(D) Lane scans of data in Figure 7A where Pol δ was absent in the pulse, and was then either present or absent during the chase.

Supplemental Tables

Supplemental table 1 | Affinity tag strategies for protein purification, related to Experimental Procedures

Protein	Yeast strain	Affinity tag strategy	Affinity tag sequence
Mrc1	yJY32	C-terminal 2xFLAG	DYKDDDGDYKDDD
Pol δ	yAE34	C-terminal TEV-CBP tag on Pol32	ENLYFQGEKRRWKKNFIAVSAANRFKKISSSGAL
RFC	yAE41	N-terminal TEV-CBP tag on RFC3	MKRRWKKNFIAVSAANRFKKISSSGALENLYFQGE
Topo I	yAE42	N-terminal TEV-CBP tag	MKRRWKKNFIAVSAANRFKKISSSGALENLYFQGE
PCNA	yAE43	N-terminal TEV-CBP tag	MKRRWKKNFIAVSAANRFKKISSSGALENLYFQGE
Csm3/Tof1	yAE48	N-terminal TEV-CBP tag on Csm3	MKRRWKKNFIAVSAANRFKKISSSGALENLYFQGE
Pol ε – Δcat	yAJ25	C-terminal TEV-CBP tag on Dpb4	ENLYFQGEKRRWKKNFIAVSAANRFKKISSSGAL

All N-terminal tags are located immediately upstream of the original start codon

All C-terminal tags are located immediately before the original stop

Supplemental table 2 | *Saccharomyces cerevisiae* strains, related to Experimental Procedures

Strain	Genotype	Reference
yAE34	*MATa ade2-1 ura3-1 his3-11,15 trp1-1 leu2-3,112 can1-100*<br>*bar1::Hyg*<br>*pep4::KanMX*<br>*ura3::URA3pRS306-POL31+POL3*<br>*his3::HIS3pRS303-Pol32-CBP+Gal4*	This study
yAE41	*MATa ade2-1 ura3-1 his3-11,15 trp1-1 leu2-3,112 can1-100*<br>*bar1::Hyg*<br>*pep4::KanMX*<br>*ura3::URA3pRS306-RFC2+CBP-RFC3*<br>*trp1::TRP1pRS304-RFC4 + RFC5*<br>*his3::HIS3pRS303-RFC1+Gal4*	This study
yAE42	*MATa ade2-1 ura3-1 his3-11,15 trp1-1 leu2-3,112 can1-100*<br>*bar1::Hyg*<br>*pep4::KanMX*<br>*his3::HIS3pRS303-CBP-TOP1+Gal4*	This study
yAE43	*MATa ade2-1 ura3-1 his3-11,15 trp1-1 leu2-3,112 can1-100*<br>*bar1::Hyg*<br>*pep4::KanMX*<br>*his3::HIS3pRS303-CBP-Pol30+Gal4*	This study
yAE48	*MATa ade2-1 ura3-1 his3-11,15 trp1-1 leu2-3,112 can1-100*<br>*bar1::Hyg*<br>*pep4::KanMX*<br>*ura3::URA3pRS306-CBP-Csm3 + Tof1*	This study
yJY32	*MATa ade2-1 ura3-1 his3-11,15 trp1-1 leu2-3,112 can1-100*<br>*bar1::Hyg*<br>*pep4::KanMX*<br>*his3::HIS3pRS303-Mrc1 C-term 2x Flag*	This study
yAJ25	*MATa ade2-1 ura3-1 his3-11,15 trp1-1 leu2-3,112 can1-100*<br>*bar1::Hyg*<br>*pep4::KanMX*<br>*ura3::URA3pRS306/Dpb2, Dpb3*<br>*trp1::TRP1pRS304/ Pol2Δ1262, Dpb4-Tev-CBP*	This study

Supplemental table 3 | Plasmids, related to Experimental Procedures

Plasmid	Original vector*	Insert	Plasmid construction
vJY17	pJF2	Mrc1-2x Flag	Synthetic construct cloned 5′ - SgrAI , 3′ - NotI
pRS306/Pol3-Gal-Pol31	pJF5	Pol3<br>Pol31	Synthetic construct cloned 5′- SgrAI , 3′- NotI<br>Synthetic construct cloned 5′- AscI , 3′- Xho1
pRS303/Pol32-CBP-Gal-Gal4	pJF2	Pol32-CBP	Synthetic construct cloned 5′- SgrAI , 3′- NotI
pRS306/RFC2-Gal-CBP-RFC3	pJF5	RFC2<br>CBP-RFC3	Synthetic construct cloned 5′- SgrAI , 3′- NotI<br>Synthetic construct cloned 5′- AscI , 3′- Xho1
pRS305/RFC5-Gal-RFC4	pJF3	RFC5<br>RFC4	Synthetic construct cloned 5′- SgrAI , 3′- NotI<br>Synthetic construct cloned 5′- AscI , 3′- Xho1
pRS303/RFC1-Gal-Gal4	pJF2	RFC1	Synthetic construct cloned 5′- SgrAI , 3′- NotI
pRS303/CBP-Pol30-Gal-Gal4	pJF2	Pol30	Synthetic construct cloned 5′- SgrAI , 3′- NotI
pRS303/CBP-Top1-Gal-Gal4	pJF2	CBP-Top1	Synthetic construct cloned 5′- SgrAI , 3′- NotI
pRS306/Tof1-Gal-CBP-Csm3	pJF5	Tof1<br>CBP-Csm3	Synthetic construct cloned 5′- SgrAI , 3′- NotI<br>Synthetic construct cloned 5′- AscI , 3′- Xho1
vJY19	pET28a	PCNA	Native PCNA gene PCR amplified from W303 genomic DNA with oligos JTY149 and JTY150. PCR product was digested with Pci1 and BamH1 and ligated into pET28a linearised with Nde1 and BamH1.
pRS306 (Dpb2 + Dpb3)	pJF19	Dpb2<br>Dpb3	Synthetic construct cloned 5′- SgrAI , 3′- NotI<br>Synthetic construct cloned 5′- AscI , 3′- Xho1
pRS304 (Δ 1262 + Dpb4-CBP)	pRS304 (Pol2 + Dpb4-CBP)	Pol2Δ1262<br>Dpb4-CBP	Synthetic construct cloned 5′- AscI , 3′- Xho1<br>Synthetic construct cloned 5′- SgrAI , 3′- NotI

Synthetic constructs were codon optimised for expression in *Saccharomyces cerevisiae* (Sharp and Li, 1987). Sequences from the 5′ and 3′ end of the PGK1 gene were added to all synthetic genes except Mrc1. Genes were synthesised by GeneArt Gene Synthesis (Life technologies).

* For details of the original expression vectors see (Coster et al., 2014; Frigola et al., 2013).

Supplemental table 4 | Oligonucleotides, related to Experimental Procedures

Oligonucleotide	Sequence
JTY107	TTCTAGAAAACCCGGGCTTTGATATGCCGGAAGGAGTTG
JTY108	TCCACTGGGTGGTACCGTGTTAGCACAATCCATATGCC
JTY161	Bio-CCGTGCCAAAGAAGAAAGTC
JTY164	GCAGGTCGACTCTAGATACGAAC
JTY149	GTGTATAAAAACATGTTGGAAGCTAAGTTCGAAGAAGC
JTY150	TTCAATTCAAGGATCCTTATTCTTCATCGTTGAACTTTGG
JTY180	GAATAATGGAAGGGTTAGAACCTACCAT
AJ34	ATAAATGGCGCGCCATGCCATCTATGGACGAAGACTA
AJ32	ATTATACTCGAGGCATAAAGGCATTAAAAGAG

Supplemental Experimental Procedures

Replication templates

An approximately 7.7 kb region surrounding ARS1 was amplified from *S. cerevisiae* W303 genomic DNA by PCR with primers JTY107 and JTY108. The PCR product was then cloned into pBluescript KS+ using the restriction enzymes Kpn I and Xma I to give the plasmid vJY22. The sequence of ARS1 was confirmed by sequencing. For soluble replication reactions covalently closed plasmid templates were purified using a Qiagen Maxi Prep Kit followed by one round of caesium chloride density gradient centrifugation.

Primed M13mp18 ssDNA was prepared by annealing the oligonucleotide JTY180 to M13mp18 single stranded DNA (New England Biolabs). Excess oligonucleotide was removed using an S-400 column (GE Healthcare).

The linear bead-coupled DNA templates used to differentiate leading and lagging strand products (Figures S1 and S6) were generated by PCR using the primers JTY161 and JTY164 with the synthetic DNA construct JY_String_1

(GeneArt) as template. DNA was coupled to Dynabeads M-280 Streptavidin (Invitrogen) as described previously (Yeeles et al., 2015).

JY_String_1

CCGTGCCAAAGAAGAAAGTCAATGACCCGTACGCCACGTACGAAACCGGTGAATCAATG
CCACACAGAGAGAAGGGAGTGGGTAAACACAGAACCCGGCGGCCAGAGGGGGGACGG
GGTAAACAGGGGAAGGAAACAAGAGAACACTGATGAACAGACACAAACCGACCAACGG
GGAAACGGCGCTCGGGCAAGACAAGCGCAGAGCAGAAAAAGAAAACCAGAAACCCAGC
AGCGGCAGGCGAAGCGAAGGACTAAGGGGACAAACCCTCACCAGAAACAAGGGAGCC
AACGCACTGAAACACAAGGGCGAACCTGGCGGTCACAACACACCCAAACAGGGGACAA
GAGCGTGGCACGAAGGCCAGAGGAACATGCAGCAACAGACCACCAGAAGAACAGAGTC
GTAGACAGAACCGAACAACCCCGCCACAGCGACAAGGAACGAAAAGGACAAAAGACCA
CCGCGAGACGTACCCACCATGAAGCCTGGGCGTATGCGCGGCGGGCCCGTCGAAAAC
AAAAAACGCAAATCCACGACACGCCGACGCGGGACGGAGAGAGAAGCCACGACCGAC
GGACAAACAAAGGAACCCGGCCGAGAGAGGAACCCGCACACACGAACACCAGAACAGA
GAGCCGAAAGATCAAAGCAGACAAGCAAACAAGAGACCCAATCAGGAACAGGCCCCGT
CAGCGGGCGACGCAACAGCACCAGGAAGACAGGGGAACCACTCAGATGGCGCACCGA
GGGCAAAACAGGGGAACCGGACAGAAAAAACGGCGCGAGGCGACGAGCCGAGAGACA
GCGACGCCAGCCCCGCGAGCAAAGGCTAATCCCCAGCACGCTACGTGTCGCCGGGGT
GAGGCAGCAAGATCTCGCGAACAACGTGGAACCAAAAAGCCTGAGGGGAGGGACGCCA
AAGCACGAAACACACGCCAAGAGGAAGGCAGCAACCTATCGACCGCAAAAGGAGAAGA
ACCCCACAGGAAAATAAAAGTCAACCAGAACCACCCTGAATGAAAAACGAAAGCACGGT
GCCGTTAACCCAAAACAACGAAGGAGGAGGGACGGAAGGGGAAAACGAAAGGCGCAC
GGAGGAAGCGGAGACAAGAAAAGCAGACGGGGCCGGGTTAGGAATATCCCCTCAGGAA
ACGAGCAGAACAGAAAAGGACAAACGGAGCCAGATCAAGTAAATAACACGGAGCATATC
AATGGAACACGGCAGCAAAAGAAGCAGCCAGAAGCCAAGATACCAGCGCCGGACCCGC
CACGCCAACTACAGGAAACCGCAGACAGAGGAGAGCCAAACCCACAGGAGAGCACAAA
ATGACACAAACGAAGGGCAAGATCGGCAAAACTAACCCGCGCACCACGGTAACGGGCG
CAAGAAGCGCCGAACCGGTTAGGTTATTACTGAGTAGTATTTATTTAAGTATTGTTTGTG
CACTTGCCTGCAGGCCTTTTGAAAAGCAAGCATAAAAGATCTAAACATAAAATCTGTAAG
AATTCCTTGTCGATTCTGGCGTTCTTTATTCCATTTGCTTTCGGTTGCGCGATCGTCCCG
ACTTTTCGCCTGAGTTTTAGGGCCCCTTTTCTTTCTTCGAGTTTCCTTCCTTTTCTCTGCT
CGCTCGTCGGCTTGTGTTTGTTCCCCGCCCAGTCGGCGCGCCACCGTTGAGGTGTTTC
CTGCGTTCCTCTCTGTTTCCTTGTCTTAATCCTTCGTTTGGATCTCCTTGTTGGGTGTTCT
TTGGGTTGGTGCTTGTTGGTGCGTTTTTTGGCTGTGGCTCCAGTTGTGTTTGGCCCGCA
TCCCTCGGTTGGCCTTCGTTTCTGGTCCGTTTCGTCGTTTTGCCGGTTGTCGTAGGTCGT
TGGGTTGGTTCTGTGTCGTGCGAGTTCCTTTCCTCCTATTCTTTTGTGGGTGCTGGCTTG
GGTAGGCGTTTTTTTCCATCGCTTTCGGCGGGTTTCGTGTCTGCCCTCGCGGTTTCATG
ATCGGCTCTCTTCGGCCCTTTCCTCTTCCGGCGGTGGATCCTCCACATGGCTCTGACGT
TGTTGGTGTGGGGGCTGCTCGGCGTCGTTGCTCCCTCGGTCCCTCCGCGGTTTTCGTT
GTGGTAGCTTCTTTCGCCAGACTCTCTTGGTCTGGTGCGGCCCTTGCCTTTTTCCTTGGT

TGCGTTCCCACTCTTCCGGCTTTCGTCTCGGCCGTTTGGGCGACTTTATGCCCCCTATT
GCGTCTTTGTCGGGTCATCCCGGTGGCCTTGTGGCTTGCGGGCTTCCCTTTCGTCGCTC
TCTTCTGGGTATTGGTCGCCCGTTTACTGTTACTTGGGGATGCTTGCCTGTGCCTGGGG
TCCTACTTTCTGGCGCTATTGGGTGCTTTTTCGGTTTTTCACAGGTCATTTTTGTGTCTGA
GTCCCTTGTGGGGTCCTCTGTCCGCGTCCCTTGCTCGTTTCGGTTCCCGCGGTCGTTTG
CCTATCCGATCGGCCTTTCTTTGCGACGTTCTTTTCTCTCTGGGGTGGTGTTGTACCCTG
TCGGCCCCTCGGGCTTTTGTTTTGACGTTGTCAGGTTTTAGTTTGGTGCGCTCGACTCTT
GGGGGGCGTGTGGGTCAGCCGTCGCTTACGCCGTGGTTTTGGAGGGCTCGGTTGTAAT
CCTTCTTGCGGCTTGTACCTTTAGGTCGTGCATCGTTTCATCTCCCTTCCGTTAAGCTGC
ATCGTGCGCGTTGAGCGTTCGGTGTTGGTCCAACTCCCTGATATTGTTTTGGCCTTCTTG
CTCTCTTCTTTCCTTTGGCTGTTTGGTTTCCGTATTGGCGTCCCCTCTCGGCGCCCTCGT
TGTCCGTCTTCTGCGCGTTTGTTTTTCGTTCACTCGGTGATGGCTCATGGGAGTTGATTG
CTCTTCTCCGCCTCCTTCTGGTCTCTCTGTTCGTA

Replication protein expression and purification

Cdt1-Mcm2-7, ORC, Cdc6, DDK, S-CDK, Sld3/7, Sld2, Dpb11, Pol ε, Cdc45, GINS, Mcm10, Ctf4, RPA, Pol α and Topo II were purified as described previously (Yeeles et al., 2015). Topo I, RFC, PCNA, Csm3/Tof1, Mrc1 and Pol δ were expressed in budding yeast (see tables 1-4 for details of strains and strain construction). Cells were grown at 30°C to 2-3 x 10^7 cells per ml in YP + 2% raffinose. Protein expression was induced by addition of galactose to 2% and cell growth was continued for 2 hours at 30°C. Cells were harvested, resuspended in lysis buffer (see specific protein purification methods for details), frozen dropwise in liquid nitrogen and crushed either in a Spex SamplePrep 6775 freezermill, or manually using a pestle and mortar cooled with liquid nitrogen. Cell powder was stored at -80°C. All subsequent protein purification steps were conducted at 4°C.

PCNA purification PCNA was purified following overexpression in both *E. coli* and yeast. No significant differences were observed between replication reactions using the *E. coli* and yeast expressed proteins.

PCNA was purified following overexpression in *E. coli* using a method modified from (Ayyagari et al., 1995). Bl21 (DE3) Rosetta, transformed with pET28a-

PCNA (supplemental table 3) (600 ml culture), were grown at 37°C in LB + 100 μg/ml ampicillin + 34 μg/ml chloramphenicol to an OD_{600} of 0.5. Protein expression was induced by addition of IPTG to 0.5 mM and growth was continued for 2.5 hours. Cells were harvested by centrifugation, washed once in 50 mM Tris-HCl pH 7.2, 10% w/v sucrose and resuspended in 20 ml 50 mM Tris-HCl pH 7.2, 10% w/v sucrose + protease inhibitors (0.3 mM PMSF, 7.5 mM Benzamidine, 0.5 mM AEBSF, 1 mM Leupeptin, 10 μg/ml Pepstatin A and 1 μg/ml Aprotinin (Sigma)). All purification steps were performed at 4°C. Cells were lysed via sonication (40%, 5s on/5s off, total 1 min) and cell debris was cleared by centrifugation (235,000g, 4°C, 30 min). Ammonium sulphate was added to 150 mM followed by addition of polymin P to 0.4% from a 10% stock. Following 10 min stirring insoluble material was removed by centrifugation (27,000g, 4°C, 15 min). To the supernatant 0.23 g/ml solid ammonium sulphate was added slowly and the sample was stirred for 10 min prior to centrifugation (48,000g, 4°C, 15 min). Insoluble material was discarded and 0.24 g/ml solid ammonium sulphate was added to the supernatant followed by 10 min stirring and centrifugation (48,000g, 4°C, 15 min). Precipitated material was resuspended in 3 ml 25 mM Tris-HCl pH 7.2, 10% glycerol, 1 mM EDTA and 100 mM NaCl (buffer P + 100 mM NaCl) and the sample dialysed against the same buffer for 1 hour. Sample conductivity was adjusted to the equivalent of buffer P + 150 mM NaCl by dilution in buffer P and was then applied to a 1 ml HiTrap SP FF column and a 1 ml HiTrap heparin column assembled in tandem. The flow through was collected and applied to a 1 ml DEAE column equilibrated in buffer P + 150 mM NaCl. Proteins were eluted with a 25 ml gradient to buffer P + 600 mM NaCl. PCNA containing fractions were pooled, diluted 2-fold in buffer P and applied to a 1 ml MonoQ column equilibrated in buffer P + 150 mM NaCl. PCNA was eluted with a 30 ml gradient to 600 mM NaCl, peak fractions were pooled, concentrated to ~ 400 μl and applied to an S200 column equilibrated in buffer P + 150 mM NaCl. Fractions containing PCNA were pooled,

frozen in liquid nitrogen and stored at -80°C. Protein concentration was determined using the Bradford assay (Bio-Rad), as was the case for all proteins used in this study.

To purify PCNA following expression in yeast, 50 g cell powder was resuspended in 120 ml buffer P + 400 mM NaCl without EDTA. Cell debris was cleared by centrifugation (235,000g, 4°C, 45 min), $CaCl_2$ was added to 2 mM and the sample was filtered through 0.45 μM syringe filters (Millipore). 1.5 ml Calmodulin Affinity Resin was added and the sample incubated for 90 min. Resin was collected, washed extensively in buffer P + 400 mM NaCl without EDTA + 2 mM $CaCl_2$ and bound proteins were eluted in buffer P + 400 mM NaCl + 2 mM EGTA. The sample was diluted in buffer P until the conductivity was equivalent to buffer P + 150 mM NaCl before separation on a 1 ml monoQ column as described for the *E. coli* expressed protein. Peak fractions were pooled and the conductivity of the sample adjusted to buffer P + 150 mM NaCl. The sample was again separated on a 1 ml monoQ as described above except that the gradient was from 150 mM NaCl to 600 mM NaCl over 30 column volumes. Peak fractions were pooled and processed as described for the protein expressed in *E. coli*.

Topo I purification. Cell powder from a 10L culture of yAE42 was diluted 2:1 in 25 mM Tris-HCl pH 7.2, 10% glycerol, 0.02% NP-40-S, 300 mM NaCl (Buffer T + 300 mM NaCl) + protease inhibitors + 1 mM DTT. Cell debris was cleared by centrifugation (235,000g, 4°C, 45 min), $CaCl_2$ was added to 2 mM and the sample was filtered through 0.45 μM syringe filters (Millipore). To the soluble extract 800 μl Calmodulin Affinity Resin (Agilent Technologies) was added and the sample incubated for 90 min. Resin was collected in a 20 ml disposable column (Bio-Rad), washed with 100 ml Buffer T + 300 mM NaCl + 2 mM $CaCl_2$ + 1 mM DTT and resuspended in 1 ml of the same buffer + 100 μg/ml TEV protease. Following a 3-

hour incubation the eluate was collected and any remaining proteins were eluted with 1 ml Buffer T + 300 mM NaCl + 2 mM $CaCl_2$. The eluates were pooled, dialysed for 3 hours against Buffer T + 300 mM NaCl and passed over an ~ 250 μl TALON column (Clontech) equilibrated in the same buffer. The flow through was collected, concentrated to ~ 500 μl and separated through an S200 column equilibrated in Buffer T + 300 mM NaCl + 1 mM DTT. Peak fractions were pooled and concentrated to ~ 1 mg/ml prior to storage.

RFC purification yAE41 cell powder (40g) was diluted ~ 2:1 in 25 mM HEPES-KOH pH 7.6, 10% glycerol, 1 mM DTT, 400 mM NaCl (Buffer R + 400 mM NaCl) + protease inhibitors. Cell debris was cleared by centrifugation (235,000g, 4°C, 1 hour) and $CaCl_2$ was added to 2 mM together with 1.5 ml Calmodulin Affinity Resin. Following 90 min incubation the resin was collected, washed with 80 ml Buffer R + 400 mM NaCl + 2 mM $CaCl_2$ and bound proteins eluted with Buffer R + 400 mM NaCl + 2 mM EDTA + 2 mM EGTA. The eluate was slowly diluted ~ 2-fold by addition of Buffer R before being applied to a 1 ml MonoS equilibrated in Buffer R + 1 mM EDTA + 200 mM NaCl. Proteins were eluted with a 30 ml gradient to Buffer R + 1 mM EDTA + 1 M NaCl. Peak fractions were pooled, concentrated to ~ 400 μl and separated through an S200 column equilibrated in Buffer R + 1 mM EDTA + 150 mM NaCl. RFC containing fractions were pooled and concentrated to ~ 2 mg/ml.

Pol δ purification Cell powder from yAE34 (60g) was thawed and diluted 2-fold in Buffer T + 400 mM NaCl + 1 mM DTT + protease inhibitors. After clearing the cell debris by centrifugation (235,000g, 4°C, 1 hour) the NaCl concentration of the lysate was reduced to 300 mM by dilution in Buffer T + 1 mM DTT + protease inhibitors. $CaCl_2$ was added to 2 mM and the sample was applied to an 800 μl Calmodulin Affinity Resin column under gravity flow. The column was washed with 100 ml Buffer

T + 1 mM DTT + 300 mM NaCl + 2 mM $CaCl_2$ and 10 ml Buffer T + 1 mM DTT + 200 mM NaCl + 2 mM $CaCl_2$ without NP-40-S. Proteins were eluted in Buffer T + 1 mM DTT + 200 mM NaCl + 2 mM EDTA + 2 mM EGTA without NP-40-S. Peak fractions were pooled and applied to a 1 ml HiTrap Heparin column equilibrated in Buffer T + 1 mM DTT + 200 mM NaCl + 1 mM EDTA without NP-40-S. Pol δ was eluted with a 30 ml gradient to 1 M NaCl. Pooled fractions were concentrated to ~ 500 μl and were separated through an S200 column equilibrated in Buffer T + 1 mM EDTA + 1 mM DTT + 150 mM NaCl. Peak fractions were pooled and concentrated prior to snap freeing in liquid nitrogen and storage at -80°C.

Csm3/Tof1 purification Cell powder form a 6 L culture of yAE48 was thawed and diluted 2:1 in Buffer T + 1 mM DTT + 200 mM NaCl. After clearing the lysate by centrifugation (235,000g, 4°C, 1 hour) $CaCl_2$ was added to 2 mM together with 500 μl Calmodulin Affinity Resin and the sample incubated for 90 min. Resin was collected in a 20 ml column (Bio-Rad) and the column washed with 50 ml Buffer T + 1 mM DTT + 200 mM NaCl + 2 mM $CaCl_2$. Resin was resuspended in 800 μl of the column wash buffer and TEV was added to 100 μg/ml. The sample was incubated on ice for 2 hours. Cleaved proteins were eluted and dialysed against 1L Buffer T + 100 mM NaCl. To remove the His-tagged TEV protease the dialysed sample was applied to an ~ 250 μl TALON column equilibrated in dialysis buffer. The flow through was then concentrated to ~ 400 μl and applied to an S200 column equilibrated in Buffer T + 150 mM NaCl. Peak fractions were pooled and concentrated to ~0.7 mg/ml.

Mrc1 purification Cell powder form a 10 L culture of yJY32 was thawed and diluted 2:1 in Buffer T + 0.5 mM DTT + 1 mM EDTA + 400 mM NaCl (Buffer M + 400 mM NaCl) + protease inhibitors. Insoluble material was cleared by centrifugation (235,000g, 4°C, 1 hour) and 1.5 ml FLAG M2 Affinity gel (Sigma) was added to the

supernatant. The sample was incubated for 90 min before the resin was collected in a 20 ml column and was washed with 40 ml Buffer T + 0.5 mM DTT + 1 mM EDTA + 400 mM NaCl. Resin was then resuspended in 10 ml Buffer T + 0.5 mM DTT + 400 mM NaCl + 10 mM magnesium acetate + 1 mM ATP and the sample incubated for 10 min. Resin was collected and washed with 40 ml Buffer T + 0.5 mM DTT + 1 mM EDTA + 400 mM NaCl. FLAG-tagged proteins were eluted by incubating the resin for 30 min in 1 ml Buffer T + 0.5 mM DTT + 1 mM EDTA + 400 mM NaCl + 0.5 mg/ml 3x FLAG peptide, and a further 10 min in buffer containing 0.25 mg/ml 3x FLAG peptide. Eluates were pooled, slowly diluted ~ 2-fold in Buffer T + 0.5 mM DTT + 1 mM EDTA and applied to a 1 ml MonoQ equilibrated in Buffer T + 0.5 mM DTT + 1 mM EDTA + 200 mM NaCl. Proteins were eluted with a 30 ml gradient to Buffer T + 1 M NaCl. Mrc1 containing fractions were pooled and dialysed for 2.5 hours against Buffer T with 40% glycerol + 0.5 mM DTT + 1 mM EDTA + 150 mM NaCl.

Pol ε – Δcat expression and purification

The codon optimised Pol2 gene (Yeeles et al., 2015) was amplified with primers AJ34 and AJ32 to remove the nucleotide sequence encoding the first 1262 amino acids. The full length Pol2 gene was then replaced with the truncated form and the resulting plasmid was used to generate the expression strain (See supplemental tables 1-4 for details). The protein was purified using a modified version of the previously published protocol for wild type Pol ε (Yeeles et al., 2015), where the heparin column was replaced by a MonoQ column. Following elution from the calmodulin affinity gel the protein was applied to a 1 ml MonoQ equilibrated in 25 mM HEPES-KOH pH 7.6, 10% glycerol, 2 mM 2-mercaptoethanol + 400 mM KOAc. Pol ε – Δcat was eluted with a 20 ml gradient from 400 mM to 1.5 M KOAc in column equilibration buffer. Peak fractions were pooled and separated through an S200 as previously described (Yeeles et al., 2015).

Bead-bound replication reactions

All reaction steps were conducted at 30°C with agitation (1200 rpm). To ensure origin specificity (Yeeles et al., 2015) ORC was pre-bound to DNA in a buffer (35 μl) containing 25 mM HEPES-KOH pH 7.6, 50 mM KCl, 10 mM $Mg(OAc)_2$, 5% Glycerol, 2 mM ATP, 1 mM DTT, 100 μg/ml BSA, 12.5 ng/μl linear DNA beads and 2.5 nM ORC. The supernatant was removed, the beads washed twice with 150 μl 40 mM HEPES-KOH pH 7.6, 10 mM $Mg(OAc)_2$, 5% glycerol, 0.02% NP-40-S and 300 mM KOAc and a new buffer (35 μl) containing 25 mM HEPES-KOH pH 7.6, 100 mM K-glutamate, 10 mM $Mg(OAc)_2$, 0.02% NP-40-S, 5% glycerol, 2 mM DTT, 5 mM ATP, 45 nM Cdc6, 100 nM Cdt1•Mcm2-7 was added and the mix incubated for 20 min. The buffer was removed and replaced with 35 μl 25 mM HEPES-KOH pH 7.6, 100 mM K-glutamate, 10 mM $Mg(OAc)_2$, 0.02% NP-40-S, 5% glycerol, 2 mM DTT, 5 mM ATP and 25 nM DDK. After a 20 min incubation the reaction was divided into three 10 μl aliquots. Replication buffer was added and reactions were initiated by addition of proteins (typically 10-15% of the final reaction volume) to give final reaction volumes of 20 μl containing 25 mM HEPES-KOH pH 7.6, 100 mM K-glutamate, 10 mM $Mg(OAc)_2$, 0.02% NP-40-S, 2.5% glycerol, 2 mM DTT, 100 μg/ml BSA, 3 mM ATP, 200 μM CTP, GTP, UTP, 80 μM dCTP, dGTP, dATP, dTTP, 12.5 nM DDK, 30 nM Dpb11, 200 nM GINS, 40 nM Cdc45, 20 nM Pol ε, 5 nM Mcm10, 20 nM S-CDK, 100 nM RPA, 20 nM Ctf4, 20 nM Pol α, 30 nM Sld3/7 and 45 nM Sld2. Either [α-^{32}P] labelled dCTP, dTTP or dATP (Perkin Elmer) were added to 40 nM to label nascent replication products.

Data analysis

All gels were scanned using a typhoon phosphorimager. Data were processed in ImageJ after converting the .gel files to 16-Bit Tiff files using the Linearize GelData command. For pulse-chase experiments the positions of the peaks were assigned

manually. Maximum product lengths were derived in GraphPad Prism by calculating and then smoothing the first derivative of lane profiles. For each experiment the product front was defined as being located at a specific value of the first derivative (typically 10-20% of the maximum value), at which point product intensity was rapidly increasing. The same first derivative value was used for all time points within an experiment. Data were fit to linear regressions and the slope of the regression was used to calculate replication rates.

Supplemental References

Ayyagari, R., Impellizzeri, K.J., Yoder, B.L., Gary, S.L., and Burgers, P.M. (1995). A mutational analysis of the yeast proliferating cell nuclear antigen indicates distinct roles in DNA replication and DNA repair. Mol Cell Biol *15*, 4420-4429.

Coster, G., Frigola, J., Beuron, F., Morris, E.P., and Diffley, J.F.X. (2014). Origin Licensing Requires ATP Binding and Hydrolysis by the MCM Replicative Helicase. Mol Cell *55*, 666-677.

Frigola, J., Remus, D., Mehanna, A., and Diffley, J.F.X. (2013). ATPase-dependent quality control of DNA replication origin licensing. Nature *495*, 339-343.

Liachko, I., Youngblood, R.A., Keich, U., and Dunham, M.J. (2013). High-resolution mapping, characterization, and optimization of autonomously replicating sequences in yeast. Genome research *23*, 698-704.

Sharp, P.M., and Li, W.H. (1987). The codon Adaptation Index--a measure of directional synonymous codon usage bias, and its potential applications. Nucleic Acids Res *15*, 1281-1295.

Yeeles, J.T., Deegan, T.D., Janska, A., Early, A., and Diffley, J.F.X. (2015). Regulated eukaryotic DNA replication origin firing with purified proteins. Nature *519*, 431-435.
