# Supplementary material for: How the Eukaryotic Replisome Achieves Rapid and Efficient DNA Replication
Source: Mol Cell. 2017 Jan 5;65(1):105–16. doi: 10.1016/j.molcel.2016.11.017 (PMC5222725; doi:10.1016/j.molcel.2016.11.017)
Supplement: Document S2. Article plus Supplemental Information [file mmc2.pdf]

Article

Molecular Cell

How the Eukaryotic Replisome Achieves Rapid and Efficient DNA Replication

Graphical Abstract

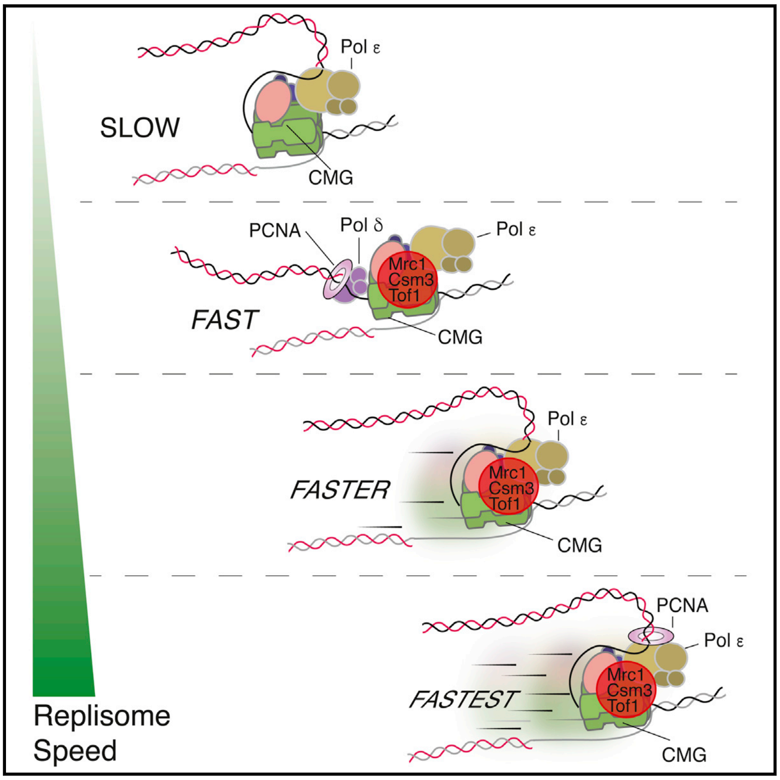

Authors

Joseph T.P. Yeeles, Agnieska Janska, Anne Early, John F.X. Diffley

Correspondence

john.diffley@crick.ac.uk

In Brief

By reconstituting a eukaryotic replisome with purified proteins that can synthesize both leading and lagging strands at the in vivo rate, Yeeles et al. reveal the basis for rapid and efficient DNA replication by the eukaryotic replisome. Maximum rates require Mrc1 and Csm3/Tof1, and they are also dependent on leading-strand synthesis by Pol ε in the presence of PCNA. Using this system the authors show that, in addition to functioning on the lagging strand, Pol δ can play an important role in establishing leading-strand synthesis before handing over to Pol ε.

Highlights

- Reconstitution of a eukaryotic replisome capable of in vivo replication rates
- Mrc1 directly stimulates replisome rate and is aided by Csm3/Tof1
- Maximum rates require leading-strand synthesis by Pol ε together with PCNA
- Pol δ plays a role in the establishment of leading-strand synthesis

Yeeles et al., 2017, Molecular Cell *65*, 105–116
January 5, 2017 © 2017 The Authors. Published by Elsevier Inc.
http://dx.doi.org/10.1016/j.molcel.2016.11.017

How the Eukaryotic Replisome Achieves Rapid and Efficient DNA Replication

Joseph T.P. Yeeles,[1,2] Agnieska Janska,[1] Anne Early,[1] and John F.X. Diffley[1,3,*]
[1]The Francis Crick Institute, Clare Hall Laboratory, South Mimms, Potters Bar, Hertfordshire EN6 3LD, UK
[2]Present address: The MRC Laboratory of Molecular Biology, Cambridge CB2 OQH, UK
[3]Lead Contact
*Correspondence: john.diffley@crick.ac.uk
http://dx.doi.org/10.1016/j.molcel.2016.11.017

SUMMARY

The eukaryotic replisome is a molecular machine that coordinates the Cdc45-MCM-GINS (CMG) replicative DNA helicase with DNA polymerases α, δ, and ε and other proteins to copy the leading- and lagging-strand templates at rates between 1 and 2 kb min^{-1}. We have now reconstituted this sophisticated machine with purified proteins, beginning with regulated CMG assembly and activation. We show that replisome-associated factors Mrc1 and Csm3/Tof1 are crucial for in vivo rates of replisome progression. Additionally, maximal rates only occur when DNA polymerase ε catalyzes leading-strand synthesis together with its processivity factor PCNA. DNA polymerase δ can support leading-strand synthesis, but at slower rates. DNA polymerase δ is required for lagging-strand synthesis, but surprisingly also plays a role in establishing leading-strand synthesis, before DNA polymerase ε engagement. We propose that switching between these DNA polymerases also contributes to leading-strand synthesis under conditions of replicative stress.

INTRODUCTION

The eukaryotic replisome organizes all of the biochemical activities required for rapid and accurate chromosome replication. Replisome assembly is a highly regulated process that begins in G1 phase of the cell cycle. The ATP-dependent motor of the replicative helicase, the MCM complex, is first loaded as an inactive double hexamer at origins (Evrin et al., 2009; Remus et al., 2009). Origin firing occurs in S phase when MCM helicase is activated. Helicase activation requires nine firing factors to convert the MCM double hexamer into two functional Cdc45-MCM-GINS (CMG) helicases (Yeeles et al., 2015). Dbf4-dependent kinase (DDK) begins the process by phosphorylating Mcm4 and 6, which leads to the binding of Sld3/7 to phosphopeptides in these subunits and subsequent recruitment of Cdc45 (Deegan et al., 2016). S-CDK phosphorylation of Sld3 and Sld2 then promotes the recruitment of Sld2, Dpb11, DNA polymerase ε (Pol ε), GINS, and Mcm10 to MCM (Tanaka et al., 2007; Yeeles et al., 2015; Zegerman and Diffley, 2007). This is the minimal set of proteins required to form the CMG and initiate template unwinding.

The firing factors Sld3/7, Sld2, and Dpb11 are required for helicase activation, but they are not thought to form part of the replisome (Gambus et al., 2006; Kanemaki and Labib, 2006; Tanaka and Araki, 2013). The fate of Mcm10 is less clear as it has been shown to travel with replication forks and interact with MCM and DNA polymerase α (Pol α) (Douglas and Diffley, 2016; Quan et al., 2015; Ricke and Bielinsky, 2004), but it does not normally co-purify with isolated CMG (Pacek et al., 2006; van Deursen et al., 2012). Pol ε is essential for helicase activation and remains associated with CMG (Sengupta et al., 2013; Tanaka and Araki, 2013). Once the CMG is assembled, many additional proteins are recruited to replication forks to form the eukaryotic replisome. These include Ctf4, Pol α, Csm3, Tof1, Mrc1, FACT, and Topo I, which are all components of the replisome progression complex (RPC), a large CMG-containing assembly that can be isolated from S phase budding yeast cells (Gambus et al., 2006). PCNA and DNA polymerase δ (Pol δ) also localize to replication forks, although they do not stably associate with the RPC (Yu et al., 2014).

Replication of both leading and lagging strands requires priming by Pol α. Multiple in vivo studies in both budding (Clausen et al., 2015; Nick McElhinny et al., 2008) and fission (Daigaku et al., 2015) yeasts, have assigned leading-strand synthesis to Pol ε and lagging-strand synthesis to Pol δ. However, the catalytic domain of Pol ε is dispensable for viability (Kesti et al., 1999), and Pol δ synthesizes the leading strand during SV40 replication (Prelich and Stillman, 1988). Consistent with this, an alternative model recently has been proposed suggesting that Pol δ synthesizes both strands at the replication fork (Johnson et al., 2015). Although Pol δ may be able to participate in leading-strand synthesis, the contexts in which it does so and the significance of its contributions remain to be elucidated.

Several complimentary approaches have been used to measure the rate of eukaryotic replication fork progression in vivo. Using the GINS complex as a proxy for replication fork location, Sekedat et al. (2010) found that the budding yeast replisome moves at a mean rate of 1.6 kb min^{-1}. This value is in good agreement with a rate of 1.9 kb min^{-1} measured using dense-isotope transfer for forks emanating from a single replication origin (Hodgson et al., 2007). DNA-combing experiments have shown the majority of replication forks in various human cell lines travel between 1 and 2 kb min^{-1}, with an average rate of 1.5 kb min^{-1} (Conti et al., 2007).

 © 2017 The Authors. Published by Elsevier Inc.
This is an open access article under the CC BY license (http://creativecommons.org/licenses/by/4.0/).

While we have learned much about eukaryotic replisome composition and function from studies both in vivo and in cell-free extracts, a eukaryotic replisome that synthesizes DNA at in vivo rates has not yet been reconstituted with purified proteins. Loading isolated CMG helicase onto a forked template together with Pol ε, RFC, PCNA, and RPA formed a minimal leading-strand replisome that replicated DNA at an average rate of 0.26 kb min^{-1} (Georgescu et al., 2014), 4- to 8-fold slower than in vivo replication fork rates. We recently have reconstituted regulated DNA replication origin firing with purified proteins from *Saccharomyces cerevisiae* (Yeeles et al., 2015). However, despite helicase activation and CMG formation occurring via the canonical initiation pathway, the minimal replisome formed in these experiments also synthesized DNA very slowly. These studies indicate that the CMG together with Pol ε is insufficient to support normal DNA replication rates, suggesting that additional replisome components are required. Until in vivo replication fork rates are recapitulated in vitro with a reconstituted eukaryotic replisome, we will not know how such rates are achieved, nor will we understand the specific roles of Pol ε and Pol δ during replication fork progression.

RESULTS

Our reconstitution of DNA replication origin firing revealed the minimum set of proteins and protein kinase targets required for MCM helicase loading and activation, CMG formation, and the initiation of DNA synthesis (Yeeles et al., 2015). Although extensive DNA synthesis was observed, there were multiple reasons to believe the replisome was incomplete and that reaction conditions were suboptimal for replication fork progression. (1) We did not include several proteins known to be crucial for replication fork progression in vivo. (2) Replication was considerably slower than in vivo replication fork rates. (3) Replication generated large (>2,000-nt) and small (∼150-nt) products. If the small products resulted from lagging-strand synthesis as we hypothesized, then replication was incomplete since they accounted for only 30% of the total synthesis.

Replication Reactions Generate Leading- and Lagging-Strand Products

To identify the template strands from which the large and small products were derived, we constructed a template to differentially label nascent leading and lagging strands. The template has a 100-bp sequence containing the ARS1 origin (Liachko et al., 2013) and an unbiased distribution of guanine and cytosine throughout. To the 5′ side of the origin, the top strand (leading-strand template) has an ∼7:1 adenine-to-thymine bias that is reversed to the 3′ side of the origin (Figure S1A). Leading strands, therefore, preferentially label with [α-^{32}P] dTTP and lagging strands label with [α-^{32}P] dATP.

Replication in the presence of [α-^{32}P] dCTP generated two classes of product: one centering around 1.4 kb and the other around 150 bases (Figure S1B, lane 1). As observed previously, large products in [α-^{32}P] dCTP accounted for approximately two-thirds of the total DNA synthesis (Figure S1C). When nascent products were labeled with [α-^{32}P] dTTP, there was an increase in incorporated label in the large products and a decrease in small products, while we observed the opposite result with [α-^{32}P] dATP (Figures S1B and S1C). Therefore, large products are primarily synthesized from the leading-strand template and small products from the lagging-strand template. Consequently, we refer to them as leading- and lagging-strand products, respectively.

Soluble Replication Reactions with Purified Proteins

We considered that template immobilization on magnetic beads might affect replication rates, so we modified our system to enable replication of soluble plasmid templates. The experimental strategy is outlined in Figure 1A. MCM is first loaded onto the 10.6-kb circular template by ORC, Cdc6, and Cdt1. Loaded MCMs are next phosphorylated by the addition of DDK. An equal volume of replication buffer is then added containing ribonucleotides, deoxyribonucleotides, and [α-^{32}P] dCTP, and replication is initiated by the addition of a master mix of proteins containing firing factors and replication proteins.

A replication time course revealed that both leading- and lagging-strand products were generated in such reactions (Figure 1B). Lagging-strand products were ∼150 nucleotides in length, comparable to those synthesized on immobilized templates (Figure S1B). Leading-strand products increased in length until ∼60 min, and they displayed a broad distribution that was centered around 4.8 kb at 120 min (Figure S2). This size is close to half the unit length of the plasmid template, consistent with near-complete bidirectional leading-strand replication. However, as with reactions on immobilized templates, lagging-strand replication still accounted for less than 50% of the replicated products, and leading-strand synthesis rates were considerably slower than fork rates measured in vivo, with full-length products only visible from 40 min (Figures 1B and 1C). We considered that these effects could be due to the absence of the processivity factor PCNA; however, addition of PCNA and its loader RFC had little effect on leading-strand products (Figures 1D and 1E). PCNA and RFC increased the lengths of lagging-strand products, but they did not generate equal amounts of leading- and lagging-strand synthesis. Unless stated otherwise, RFC and PCNA were included in all subsequent reactions.

Reconstitution of In Vivo Replication Rates with Purified Proteins

Multiple additional proteins associate with the CMG to form the RPC (Gambus et al., 2006). We expressed and purified components of the RPC to assess their effects on replication (Figure 2A). The addition of Mrc1, FACT, Topo I, and a complex of Csm3 and Tof1 appeared to increase the rate of leading-strand synthesis dramatically (Figure 2B), with full-length products appearing within 10 min. To assess replication rates, we performed pulse-chase experiments in which the extension of products labeled in the first few minutes is followed independently of initiation kinetics (Figures 2C and 2D). The rates of leading-strand synthesis were determined by plotting both the maximum and peak product lengths at each time point and fitting to linear regressions. Using this method, we calculated the maximum elongation rate to be 1.92 kb min^{-1} and the bulk rate to be 1.44 kb min^{-1}, similar to replication fork rates that have been measured in vivo (Conti et al., 2007; Hodgson et al., 2007; Sekedat et al., 2010).

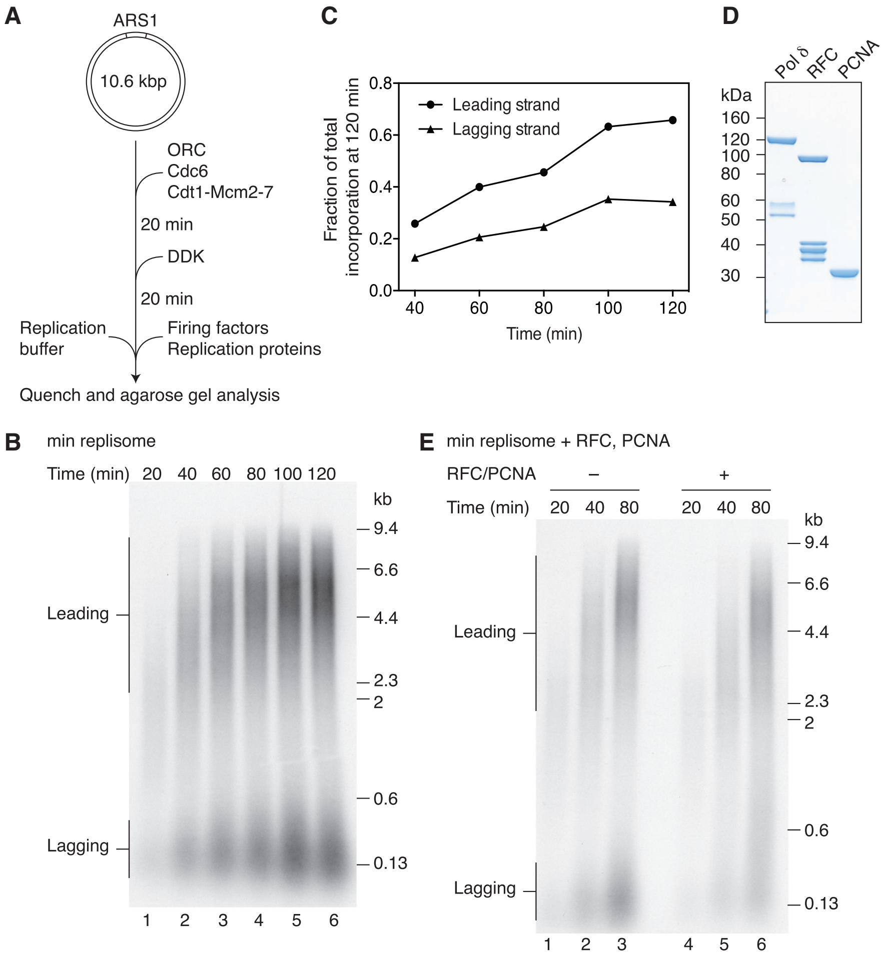

Figure 1. Replication Reactions on Soluble Plasmid Templates

(A) Reaction scheme for soluble replication reactions is shown. Firing factors: Sld3/7, Sld2, Dpb11, S-CDK, GINS, Cdc45, Pol ε, Mcm10. Replication proteins: Topo II, Pol α, RPA, Ctf4.

(B) Time course of a reaction performed as in (A) is shown.

(C) Quantitation of leading- and lagging-strand products in (B) is shown.

(D) Coomassie-stained SDS-PAGE of proteins involved in lagging-strand replication is shown.

(E) Replication performed as in (B) is shown.

In this and all subsequent figures, the protein constituents of the reactions are listed above each figure. Min replisome encompasses the minimum set of proteins required for origin firing together with Ctf4 and Topo II (see A for details).

Csm3/Tof1 and Mrc1 Are Required for Maximum Replication Rates

We left out individual RPC components, and we examined nascent DNA products at an early time point (15 min) to identify the protein(s) responsible for the increased replication rates (Figure 3A). Omission of FACT (Figures 2C and 3A, lane 5), Ctf4 (Figure S3), or Topo I (Figure 3A, lane 6) had no appreciable effect (all reactions also contained Topo II). Omitting Mrc1 significantly reduced DNA synthesis, generating a replication profile virtually identical to that of the minimal replisome (Figure 3A, lanes 1 and 3). Intermediate length products were generated when Csm3/Tof1 was left out (Figure 3A, lane 4). These observations indicate that the key RPC components for maximum replication rate are Mrc1 and Csm3/Tof1. Moreover, they demonstrate that Mrc1 can increase the replication rate of the minimal replisome without Csm3/Tof1 but that both Mrc1 and Csm3/Tof1 are required for maximum replication rates.

That Mrc1 can affect replication rate without Csm3/Tof1 but not vice versa might suggest Csm3/Tof1 acts by promoting Mrc1 function in some way. If true, the reduced replication rates observed in the absence of Csm3/Tof1 might be overcome by increasing Mrc1 concentration. Indeed, as shown in Figure 3B, maximum leading-strand length at 15 min in the presence of Csm3/Tof1 was seen even at the lowest Mrc1 concentration tested (5 nM), whereas leading-strand product length in the absence of Csm3/Tof1 increased between 5 and 15 nM Mrc1 (Figure 3B, lanes 2–4). Even at the highest concentration of Mrc1 tested (20 nM), leading strands in the absence of Csm3/Tof1 were shorter than they were in the presence of Csm3/Tof1. To test further the hypothesis that Csm3/Tof1

CellPress

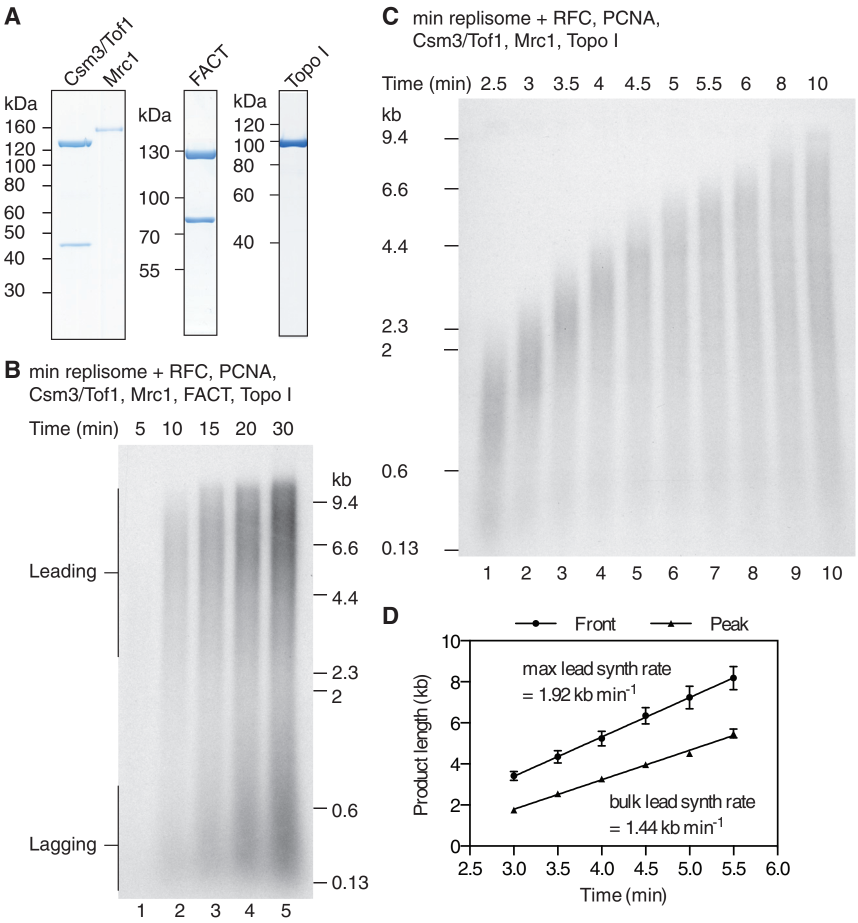

Figure 2. Reconstitution of In Vivo Replication Rates with Purified Proteins

(A) Coomassie-stained SDS-PAGE of RPC components is shown.

(B) Replication time course conducted as in Figures 1A and 1B but including RFC, PCNA, and the additional RPC components shown in (A). The additional proteins were added together with the firing factors and replication proteins.

(C) Pulse-chase experiment to measure replication rates in the presence of RPC components. FACT and Topo II were omitted. The chase was added at 2 min 20 s.

(D) Maximum (front) and peak product lengths plotted against time for pulse-chase experiments performed as in (C). Error bars represent the SEM from two experiments. Data were fit to a linear regression to derive the maximum and bulk leading-strand synthesis rates.

functionally stabilizes Mrc1, we investigated the response of the replisome to increasing salt concentrations. In reactions containing Csm3/Tof1, leading-strand synthesis was largely insensitive to increasing salt, while there was a small increase in the size of lagging-strand products (Figure 3C, lanes 5–8). By contrast, increased salt severely inhibited replication when Csm3/Tof1 was omitted. At the highest salt concentration tested, replication products resembled those generated in reactions lacking Mrc1 (compare Figure 3B, lane 1 and Figure 3C, lane 4).

Taken together, these results indicate that Mrc1 is chiefly responsible for the increased rate of synthesis and that Csm3/Tof1 acts by promoting stable functioning of Mrc1 in the replisome. They do not exclude an additional, Mrc1-independent role for Csm3/Tof1 in promoting rapid replication rates. Reaction buffers in all subsequent experiments contained 250 mM potassium glutamate, which enforced a strict dependence on Csm3/Tof1 for maximum synthesis rates (Figure 3C, lanes 4 and 8).

PCNA Functions with Pol ε during Rapid Leading-Strand Synthesis

Our experiments with the minimal replisome (Figure 1E) and those using purified CMG with Pol ε (Georgescu et al., 2014) showed that PCNA has little effect on leading-strand synthesis by Pol ε when replication was slow. However, in contrast to these more minimal systems, omission of PCNA appeared to reduce the rate of leading-strand synthesis in a time course when Mrc1 and Csm3/Tof1 were present (Figure 4A, lanes 2 and 7; Figure S4A). Similar results were seen when the clamp loader RFC was omitted (Figure S4B), and this apparent reduction in rate occurred over a range of salt concentrations (Figure S4B).

To quantify replication rates, we again used the pulse-chase protocol. In contrast to the pulse-chase with PCNA (Figure 2C), two distinct populations of products were generated in the absence of PCNA (Figure 4B). One population was efficiently extended throughout the time course, which we used to derive a maximum synthesis rate of 1.25 kb min^{-1} (Figure 4C), 35% slower than the 1.92 kb min measured in the presence of PCNA (Figure 2D) and consistent with the results from the time course (Figure 4A). The second population was extended to ~1–2 kb at 5.5 min, after which point little further extension occurred. This population of products was not observed under our standard reaction conditions in the absence of PCNA (Figure 4A; Figures S4A and S4B), suggesting that it may have arisen due to the altered deoxyribonucleoside triphosphate (dNTP) concentrations used in pulse-chase experiments. To test this idea further, we examined the effect of dNTP concentration on synthesis in the absence and presence of PCNA. In the presence of PCNA, replication was largely insensitive to dNTP concentration and rapid synthesis was observed at dNTP concentrations as low as 5 μM (Figure 4D, lanes 6–10). When PCNA was omitted, however, replication became extremely sensitive to dNTP concentration: synthesis was reduced at all concentrations below 40 μM, and, at 5 μM dNTP, we did not observe products longer than 600 nucleotides. Taken together the data reveal that PCNA is required for maximal replication rates even at standard dNTP

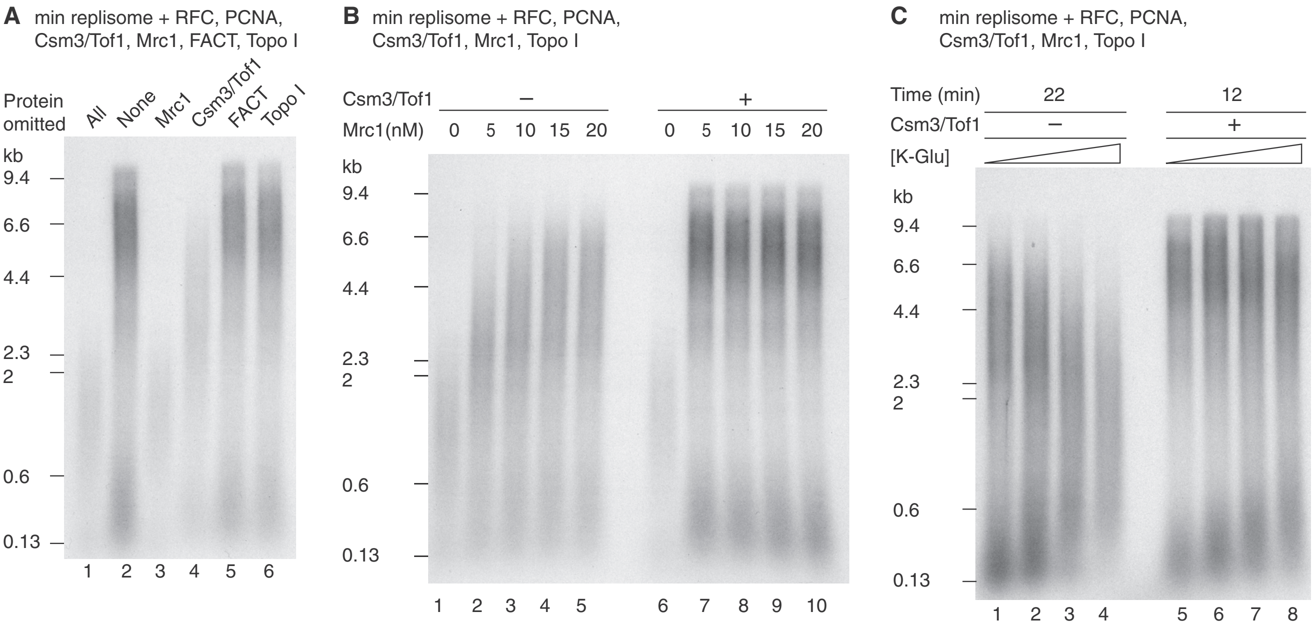

Figure 3. Csm3/Tof1 and Mrc1 Are Required for Maximum Rates

(A) Replication reactions performed with the proteins illustrated. In lane 1, All refers to Csm3/Tof1, Mrc1, FACT, and Topo I.

(B and C) Reactions were performed as in (A) except that FACT was omitted. (A) and (B) were incubated for 15 min. The potassium glutamate concentrations in (C) were 100, 150, 200, and 250 mM.

concentrations and plays an important role in leading-strand synthesis at low nucleotide concentrations.

Pol δ Is Dispensable for Maximum Synthesis Rates but Required for Complete Lagging-Strand Replication

Although Csm3/Tof1 and Mrc1 promoted rapid leading-strand synthesis, lagging-strand products were still underrepresented. We therefore examined the effect of adding Pol δ to our reactions, because multiple studies have indicated it to be the major lagging-strand polymerase in vivo. The addition of Pol δ increased the intensity of lagging-strand products (Figures 5A and 5B), and quantification of products showed that Pol δ promoted equal synthesis on both template strands (Figure 5C). Under these conditions, we found that the length of lagging-strand products was dependent upon the concentration of Pol α (Figure 5D) and that Pol δ was required for maximum lagging-strand synthesis over a range of Pol α concentrations (Figures S5A–S5C). Moreover, both RFC and PCNA were required for Pol δ to promote equal synthesis on both strands (Figures S5D and S5E).

In addition to the clear effects on lagging-strand products, we also observed subtle changes to leading-strand synthesis. First, there was a small (<15%) but reproducible reduction in the rate of leading-strand synthesis (Figures 5E and 5F). Second, there was a change in the distribution of leading-strand products with a more prominent and symmetrical peak at ~5 kb, roughly half-plasmid length (Figure 5G). Third, we routinely saw increased overall nucleotide incorporation in pulse-chase experiments containing Pol δ (Figure 5E). These observations indicate that Pol δ plays some role in leading-strand synthesis. We address this in the next sections.

Pol δ Can Function as the Leading-Strand Polymerase but Does so at a Reduced Rate

Pol ε is essential for CMG activation (Yeeles et al., 2015), and, hence, it cannot simply be omitted to enable measurement of Pol δ-catalyzed leading-strand synthesis. However, the DNA polymerase catalytic domain of the Pol2 subunit of Pol ε is dispensable for cell viability (Kesti et al., 1999), suggesting that the remainder of the protein can support initiation. We purified Pol ε lacking the catalytic domain of the Pol2 subunit, Pol ε-Δcat. This protein could, indeed, support initiation and both leading- and lagging-strand synthesis (Figures S6A and S6B). In soluble replication reactions, synthesis of both strands was now almost entirely dependent on Pol δ (Figure 6A, lanes 3 and 4). The small amount of short products in the absence of Pol δ (Figure 6A, lane 3) shows that Pol α cannot by itself support efficient leading-strand synthesis under these conditions. After 15 min, leading-strand products synthesized by Pol δ with the Pol ε-Δcat replisome were considerably shorter than those synthesized in reactions containing Pol ε, suggesting that replication was slower in the absence of the Pol ε catalytic domain (Figure 6A, lanes 2 and 4). Omission of Mrc1 from reactions with Pol ε-Δcat resulted in a further reduction in product length, illustrating that Mrc1 accelerates leading-strand replication irrespective of whether it is catalyzed by Pol ε or Pol δ (Figure 6B). This experiment also shows that leading-strand synthesis by the complete replisome with Pol δ (Figure 6B, lane 4) is still faster than synthesis by the minimal replisome lacking Mrc1, even with Pol ε catalyzing leading-strand synthesis (Figure 6B, lane 1).

The maximum leading-strand synthesis rate generated by Pol δ with the Pol ε-Δcat replisome was over 3-fold slower

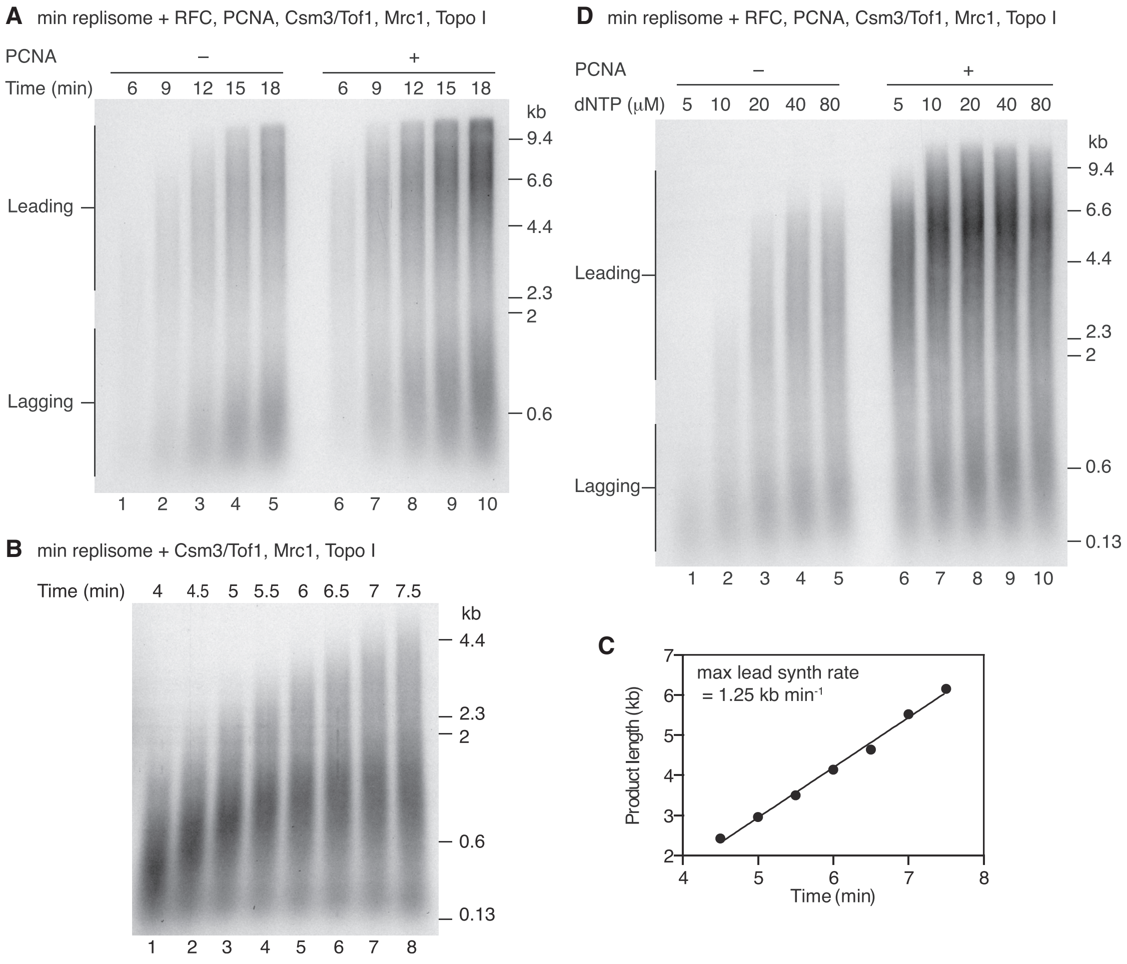

Figure 4. PCNA Has a Major Role in Pol ε-Catalyzed Leading-Strand Synthesis

(A) Time course reaction performed using the same experimental conditions as Figure 3C, lane 8. PCNA was omitted where indicated.

(B) Pulse-chase experiment performed with the same compliment of proteins as in (A) except that RFC, PCNA, and Topo II were omitted. The chase was added at 3 min 50 s.

(C) Maximum product lengths plotted against time for the pulse-chase experiment in (B). Data were fit to a linear regression to derive the maximum leading-strand synthesis rate.

(D) Experiment performed as in (A) for 8 min. The dNTP concentrations are the concentrations of the individual dNTPs in the reaction.

than Pol ε alone (Figures 2C, 2D, and 6C; Figure S6C), indicating that Pol δ can catalyze leading-strand synthesis in the absence of the Pol ε catalytic domain but that this synthesis is slower than synthesis catalyzed by Pol ε. This result, together with the fact that the presence of Pol δ only modestly reduces maximum synthesis rates with full-length Pol ε (Figures 5F and 5G), strongly suggests that Pol ε catalyzes the bulk of leading-strand synthesis, even when Pol δ is present. Leading-strand replication by Pol δ with the Pol ε-Δcat replisome could be slow because the intrinsic rate of polymerization by Pol δ was limiting in our system, or because the catalytic domain of Pol ε is required for maximum CMG-unwinding rates. We therefore measured the rate of polymerization by Pol δ under our replication reaction conditions using a singly primed M13 single-stranded DNA (ssDNA) template (Figures 6D and 6E). Replication products were elongated at over 4 kb min^{-1}, twice the maximum fork rate and over six times faster than the rate observed with the Pol ε-Δcat replisome. Given how fast Pol δ can synthesize DNA on primed ssDNA templates, it is highly likely that the reduced rate of the Pol ε-Δcat replisome therefore arises because of a reduced rate of unwinding by CMG.

Given the slower rate of leading-strand synthesis with Pol δ and the Pol ε-Δcat replisome, we reasoned that the slightly slower rate of leading-strand synthesis with Pol δ and Pol ε compared to Pol ε alone (compare Figures 2C and 2D with Figures 5E and 5F) might reflect competition between Pol δ and Pol ε for leading-strand synthesis. We therefore tested whether further increasing concentrations of Pol δ might reduce the net rate of synthesis in a reaction containing entirely wild-type DNA polymerases. Figure 6F and Figures S7A and S7B show that Pol δ did indeed slow the rate of leading-strand elongation in a concentration-dependent manner, though relatively high concentrations of Pol δ were required for

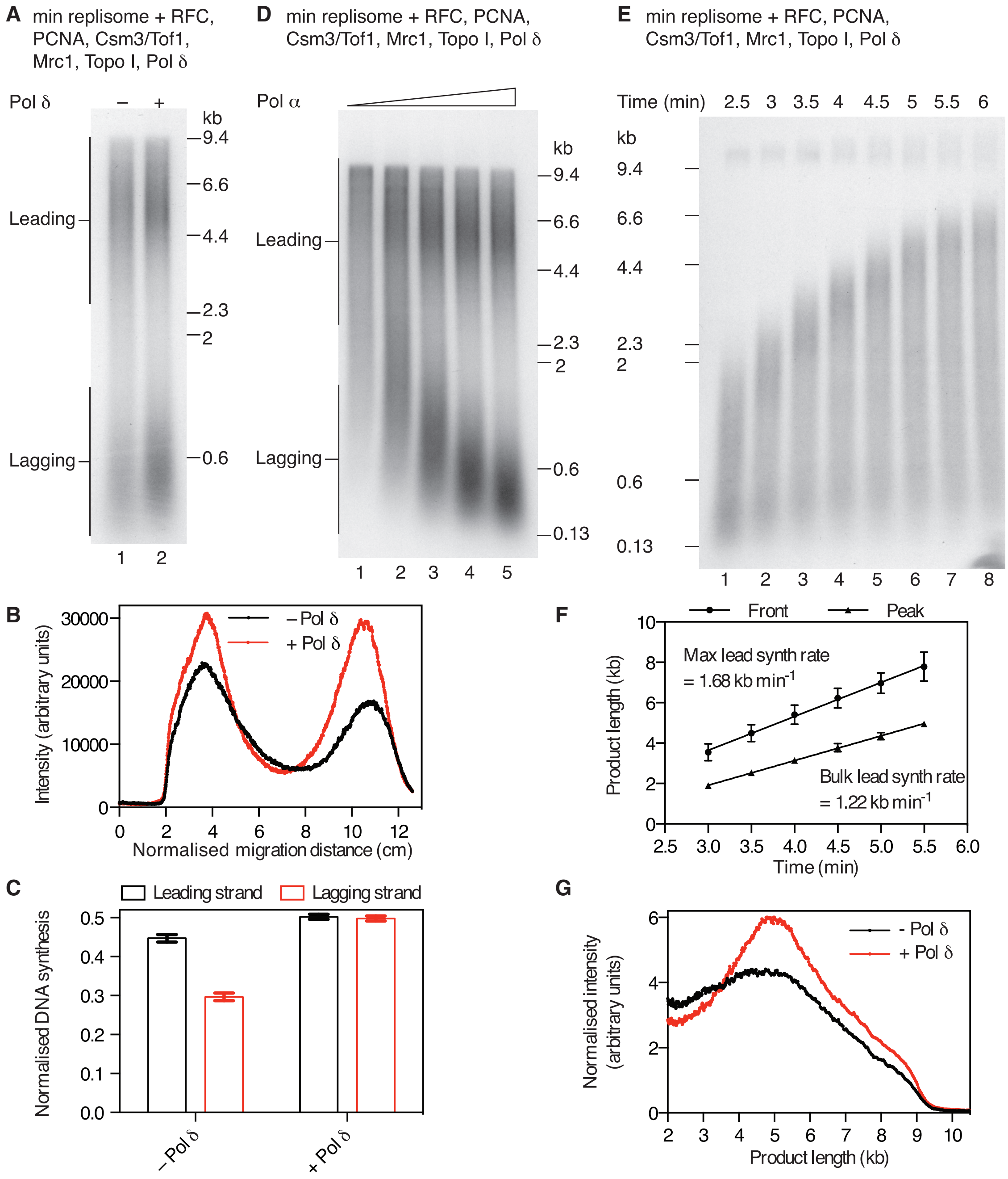

Figure 5. The Effect of Pol δ on Replication

(A) A 20-min reaction performed with the same set of proteins as Figure 4A with PCNA. The Pol α concentration was 40 nM and the Pol δ concentration was 10 nM.

(B) Lane profiles of the data in (A) are shown.

(C) Quantitation of leading- and lagging-strand replication products for experiments performed as in (A). Data were normalized to the sum of leading and lagging strands in the reaction containing Pol δ. Error bars represent the SEM from two experiments.

(D) Reaction performed as in (A) with 10 nM Pol δ for 20 min. Pol α concentrations were 5, 10, 20, 40, and 80 nM.

(E and F) Pulse-chase experiment (E) was performed and analyzed (F) as in Figures 2C and 2D but with the inclusion of 10 nM Pol δ.

(G) Normalized product-length distribution for the leading-strand products in (A). To account for the continuous incorporation of radiolabel, product intensities were divided by product lengths.

substantial inhibition. Taken together, these results show that leading-stand replication by Pol δ is inherently slower than replication by Pol ε: in the absence of any Pol ε catalysis (Pol ε-Δcat), this rate is reduced to approximately one-third the rate with Pol ε. Pol δ also can compete with Pol ε and subsequently slow leading-strand synthesis, but Pol ε is the preferred polymerase because

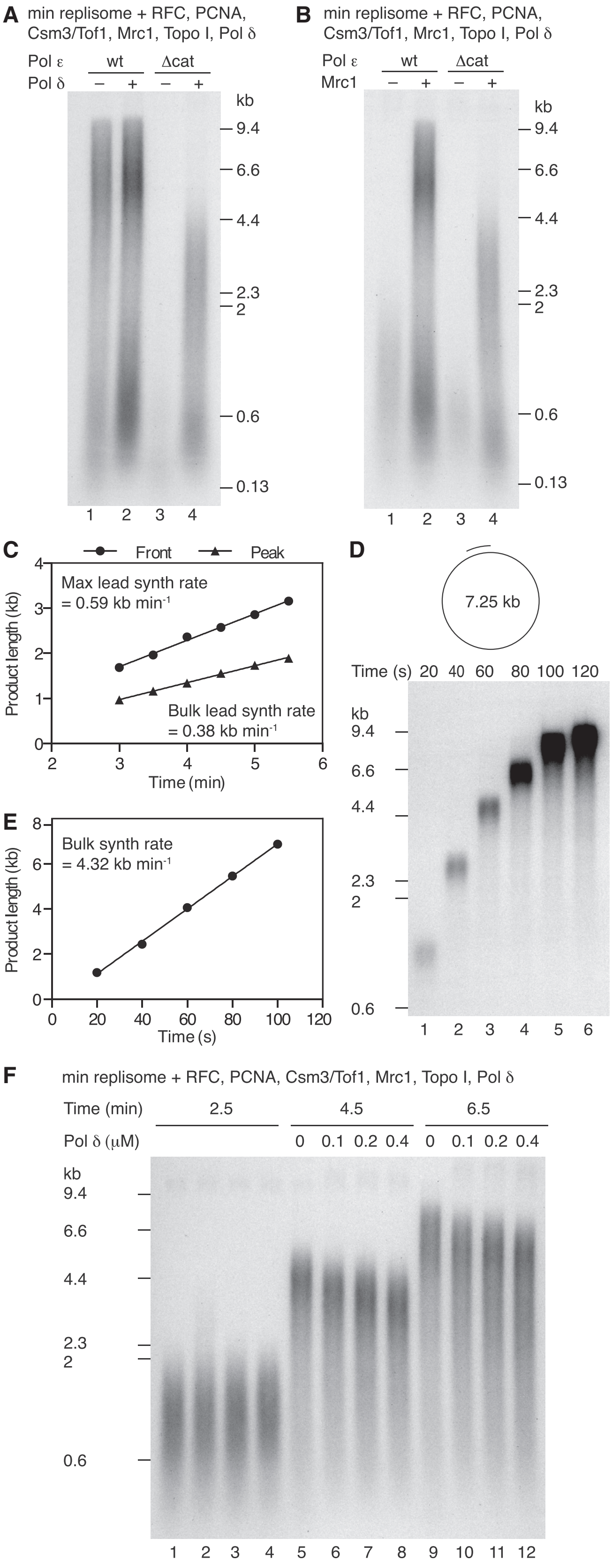

Figure 6. Leading-Strand Synthesis Catalyzed by Pol δ Is Slow

(A and B) The roles of Pol δ (A and B) and Mrc1 (B) in replication with Pol ε-Δcat. Reactions performed for 15 min with the same set of proteins as in Figure 4A with PCNA. Where indicated, wild-type Pol ε was substituted with Pol ε-Δcat.

(C) Quantitation of a pulse-chase reaction performed as in Figure 5E except that Pol ε was substituted with Pol ε-Δcat Figure S6C. The chase was added at 2 min 50 s.

(D) Primer extension reaction with Pol δ. The primed template was incubated with PCNA and RFC for 5 min before reactions were initiated by the addition of Pol δ.

(E) Quantitation of the data in (D) plotting the peak of the product distributions. Data were fit to a linear regression.

(F) Pulse-chase reactions were performed as in Figure 2C but with varying concentrations of Pol δ added immediately after the 2-min 30-s time point.

relatively high Pol δ concentrations are required to slow replication. We conclude that maximal unwinding rate by the replicative helicase and therefore maximal rate of leading-strand replication require catalysis of leading-strand synthesis by Pol ε.

Pol δ Promotes the Establishment of Leading-Strand Synthesis

The increased incorporation in pulse-chase experiments and the more symmetrical distribution of leading-strand products led us to consider that Pol δ may be acting early in reactions to elongate primers synthesized by Pol α. To examine this, we performed a pulse-chase in which Pol δ was either present in the pulse, present in the chase, or entirely absent. When Pol δ was included in the pulse, there was an increase in the amount of synthesis, but not the length, of products after 2.5 min when compared to reactions lacking Pol δ (Figure 7A, lanes 1–3). During the chase, this Pol δ-dependent increase in synthesis was translated into a greater abundance of long leading-strand products; however, the rate of leading-strand elongation was not affected whether Pol δ was present or absent (Figure S7D), indicating that Pol δ acts by stimulating the establishment of leading strands early in the reaction.

Figure 6F and Figures S7A and S7B showed that high concentrations of Pol δ can reduce the net rate of leading-strand synthesis, presumably by competing with Pol ε for the 3′ end of the leading strand. However, the apparent reduction in leading-strand rate was modest despite a 20-fold excess of Pol δ over Pol ε. Leading-strand synthesis by Pol ε is, therefore, highly resistant to challenge by Pol δ. We asked if having a 20-fold excess of Pol δ from the beginning of reactions would prevent Pol ε accessing the 3′ end of the leading strand after it had been initiated by Pol δ, but there was no further inhibition of leading-strand elongation compared to when Pol δ was added with the chase (Figure S7C). We conclude that Pol δ acts early to promote the establishment of leading-strand synthesis before handing over the leading strand to Pol ε.

DISCUSSION

We have reconstituted a eukaryotic replisome with the capacity to replicate the leading and lagging strands at rates comparable to those observed in vivo. In addition to the 14 purified proteins required for MCM helicase loading and activation (Yeeles et al., 2015), this replisome requires five additional proteins comprising

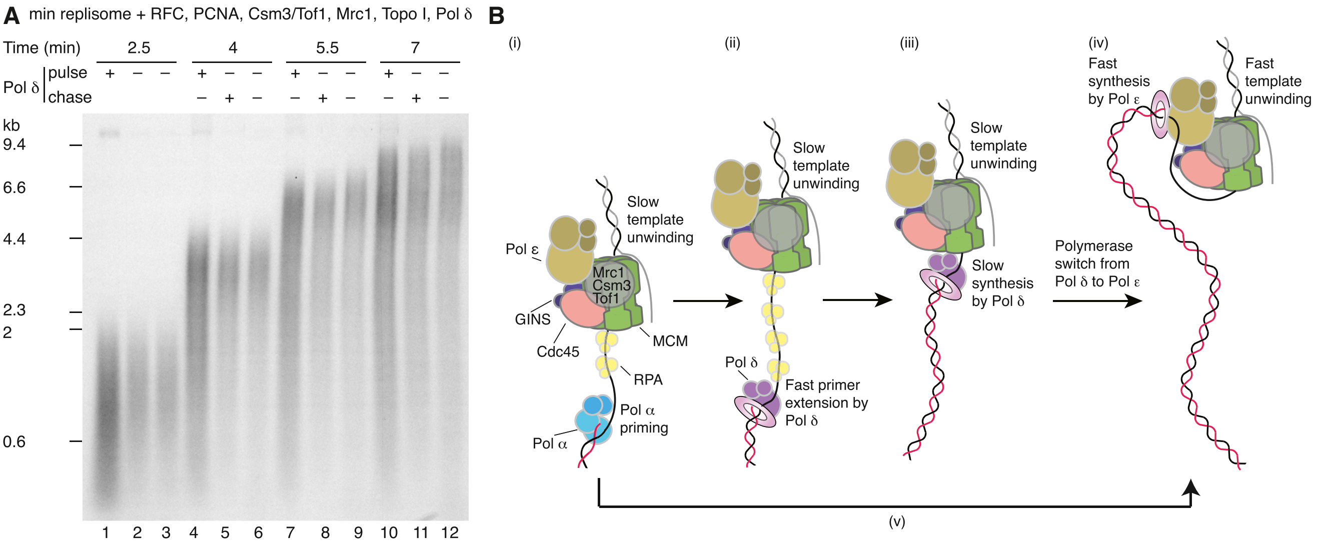

Figure 7. Pol δ Promotes the Establishment of Leading-Strand Synthesis

(A) Pulse-chase reactions conducted as in Figure 2C with 10 nM Pol δ included where indicated. When Pol δ was included in the chase, it was added immediately after the 2-min 30-s time point was removed.

(B) Model for eukaryotic leading-strand synthesis. (i) Following helicase activation the replisome advances slowly, unwinding the template to generate a priming site on the leading strand for Pol α. (ii) Following priming, RFC assembles PCNA around the primer terminus. Pol δ rapidly binds to the primer and commences elongation. The elongation rate of Pol δ is considerably faster than the advancing replisome, so Pol δ quickly catches up with the replication fork. (iii) Once Pol δ has made contact with the replisome, the rate of synthesis is limited by the template-unwinding rate of the replisome. (iv) A polymerase switch transfers the 3′ end of the leading strand together with PCNA from Pol δ to Pol ε. Pol ε-dependent leading-strand synthesis stimulates the template-unwinding rate of the replisome, and DNA synthesis rates of ~2 kb min^{-1} are established. (v) In the absence of Pol δ, Pol ε can take over leading-strand synthesis directly from Pol α, although this process is less efficient than the pathway involving Pol δ.

an additional 12 gene products: the RPC components Csm3/Tof1 and Mrc1, the processivity factor PCNA together with its loader RFC, and Pol δ. Based on the work described here, we propose a model for leading-strand replication in eukaryotes with three novel features (Figure 7B) as follows: (1) the RPC component Mrc1 acts to stimulate replisome rates directly, aided by Csm3/Tof1; (2) PCNA plays a crucial role in leading-strand synthesis with Pol ε; and (3) Pol δ can play an important role in the establishment of leading-strand replication before handing synthesis over to Pol ε.

Csm3/Tof1 and Mrc1 Are Essential for Normal Replication Rates

Mrc1 and its vertebrate homolog Claspin influence replication fork rate in cells (Hodgson et al., 2007; Petermann et al., 2008; Szyjka et al., 2005; Tourrière et al., 2005); however, the mechanisms by which they do so are unknown. Our data show that Mrc1 directly stimulates the rate of replisome progression on naked DNA. We propose that Mrc1 is principally responsible for increasing the rate of the minimal replisome, with Csm3/Tof1 acting primarily to promote the proper functional association of Mrc1 with the replisome. This conclusion, based on data in Figures 3B and 3C, is consistent with chromatin immunoprecipitation (ChIP) experiments showing that Csm3 and Tof1 are required for physical association of Mrc1 with sites of DNA synthesis (Bando et al., 2009). In vivo, *TOF1* deletion was reported to reduce fork rates to the extent seen in *mrc1Δ* cells when measured by DNA fiber analysis (Tourrière et al., 2005) but to a far lesser extent when dense isotope transfer was used to measure fork rate (Hodgson et al., 2007). The reason for these discrepancies is unclear, but it may reflect the fact that replication rates in the absence of Tof1 are sensitive to Mrc1 concentration (Figure 3B), which may be affected in vivo by strain background or environmental factors. Csm3 and Tof1 are also important for programmed replication fork pausing (Dalgaard and Klar, 2000; Krings and Bastia, 2004). The mechanism by which they promote pausing is, however, likely to be distinct from their role in facilitating normal replication fork progression, because fork pausing is not dependent on Mrc1 (Calzada et al., 2005; Hodgson et al., 2007; Mohanty et al., 2006; Tourrière et al., 2005).

It is likely that Mrc1 accelerates the replisome by directly accelerating the rate of unwinding by CMG. This would be consistent with the fact that Mrc1 increases synthesis rates regardless of whether Pol ε or Pol δ catalyzes leading-strand synthesis. In addition to Mrc1, the maximum leading-strand synthesis rate only occurs when Pol ε synthesizes the leading strand with PCNA. Leading-strand synthesis by Pol ε is faster than Pol δ even in the absence of Mrc1 (Figure 6B, lanes 1 and 3), suggesting that Mrc1 and Pol ε may act separately and additively to accelerate unwinding. However, Mrc1 interacts with both MCM and Pol ε (Komata et al., 2009; Lou et al., 2008), so it is possible that Mrc1 and Pol ε also may act together to modulate unwinding. It remains to be seen whether any of the firing factors present in our reactions like Mcm10 contribute to maximum synthesis rates. Although these factors are not required for CMG

helicase activity (Moyer et al., 2006) or replication by Pol ε with the CMG (Georgescu et al., 2014), they could be required with Csm3/Tof1 and Mrc1 for maximum replication rates.

Pol δ Is Required for Lagging-Strand Synthesis

We found that balanced leading- and lagging-strand synthesis was observed only in reactions containing Pol δ. This suggests that Pol ε cannot function efficiently on the lagging strand, in agreement with previous in vivo and in vitro results (Georgescu et al., 2015; Nick McElhinny et al., 2008). Processive synthesis by Pol δ requires PCNA (Chilkova et al., 2007), and we found efficient lagging-strand synthesis also required both PCNA and RFC. In the presence of Pol δ and PCNA, lagging-strand product length was dependent upon the concentration of Pol α, indicating that Pol α functions distributively, even in the presence of Ctf4 and Mcm10, two factors proposed to link Pol α to CMG (Gambus et al., 2009; Ricke and Bielinsky, 2004; Tanaka et al., 2009). Even at the highest concentration of Pol α tested, lagging-strand products were longer than the ~165 nucleotides that have been measured in vivo (Smith and Whitehouse, 2012). These experiments were conducted on naked DNA templates, and in the accompanying manuscript, we show that chromatin profoundly affects lagging-strand product sizes (Kurat et al., 2016).

PCNA Is Crucial for Leading-Strand Synthesis

In addition to the anticipated role of PCNA in lagging-strand replication with Pol δ, we discovered that PCNA plays a major role in leading-strand synthesis catalyzed by Pol ε. Specifically, PCNA promotes maximum replication rates, and it also is critical for rapid synthesis at low nucleotide concentrations, conditions that mimic those generated following treatment of cells with hydroxyurea. That PCNA is essential for maximum leading-strand rates might seem surprising because CMG functions as a processivity factor for Pol ε by tethering it to the replication fork (Langston et al., 2014), potentially obviating any need for PCNA. Moreover, PCNA had little effect on replication with slower replisomes in vitro (Figure 1E; Georgescu et al., 2014). We propose that Pol ε utilizes both CMG and PCNA as processivity factors to facilitate normal replication rates: CMG tethers Pol ε to the unwinding fork while PCNA promotes continued association of Pol ε to the 3′ end of the leading strand. The repeated cycling of the Pol ε catalytic domain on and off the 3′ end of the leading strand in the absence of PCNA may slow the net rate of synthesis by slowing CMG, consistent with our proposal that the rate of unwinding by CMG is maximal only when the catalytic domain of Pol ε is engaged in synthesis. Without PCNA, leading-strand synthesis is ~1.2 kb min^{-1}, still much faster than leading-strand synthesis without Mrc1 and Csm3/Tof1, which explains why PCNA doesn't affect the rate of synthesis with the minimal replisome (Figure 1E).

We suggest that PCNA on the leading strand helps prevent uncoupling of unwinding from leading-strand DNA synthesis by forming a PCNA-Pol ε-CMG bridge between the 3′ end of the leading strand and the unwinding replication fork. Katou et al. (2003) showed that, in the absence of Mrc1, CMG and Pol ε continue to progress even when DNA synthesis is inhibited with hydroxyurea, suggesting an uncoupling of unwinding from DNA synthesis. It may be that Mrc1 and PCNA play separate, distinct roles in preventing uncoupling. Alternatively, PCNA may not be loaded efficiently on the leading strand without Mrc1; perhaps Pol ε binds directly to Pol α-synthesized primers in slow-moving forks lacking Mrc1 and inhibits PCNA loading. This would be consistent with the fact that PCNA does not affect DNA synthesis in the absence of Mrc1 (Figure 1E) and consistent with the hypersensitivity of DNA synthesis to low dNTP concentration in the absence of PCNA (Figure 4D). In this regard, some Pol ε PIP box mutants, which are likely to be defective in PCNA binding, are sensitive to the alkylating agent methyl methanesulfonate (Dua et al., 2002), providing a potential link between the Pol ε-PCNA interaction and the replisome's ability to overcome DNA damage.

A Polymerase Switch Mechanism for the Establishment of Leading-Strand Replication

Our experiments suggest Pol δ can play an important role in establishing leading-strand synthesis. In the absence of Pol δ, initiation of leading-strand synthesis is compromised (Figure 7A). Moreover, the distribution of leading-strand lengths in the absence of Pol δ is broader (Figure 5), suggesting more unidirectional forks or asymmetric initiation of the two forks. We propose the model in Figure 7B to explain the role of Pol δ in leading-strand synthesis. After helicase activation, Pol α synthesizes the primer for the leading strand. While this is happening, CMG-Pol ε begins unwinding DNA at a relatively slow rate. In the absence of Pol δ, Pol ε can take over leading-strand synthesis directly, leading to fast unwinding and fast Pol ε-dependent synthesis, but this is less than completely efficient, perhaps because CMG-Pol ε is moving away from the origin while the primer is being made. In this situation, Pol δ with PCNA will take over the 3′ end generated by Pol α. Initially, because CMG-Pol ε has unwound away from the primer end, synthesis by Pol δ will be fast, as in Figure 6D; but, when Pol δ reaches the slow-moving CMG, it will slow down, as in Figure 6A, because it cannot accelerate the rate of CMG unwinding like Pol ε. At this point, we propose a polymerase switch occurs in which the 3′ end, perhaps together with the loaded PCNA, is transferred from Pol δ to Pol ε. Leading-strand synthesis by Pol ε then stimulates unwinding by CMG, promoting the maximal rates of leading-strand synthesis. This model has support from polymerase usage sequencing data (Daigaku et al., 2015), which found a bias toward Pol δ usage proximal to efficient replication origins that declined further into replicons.

While our work has revealed a specific role for Pol δ in the establishment of leading-strand replication, we hypothesize this may reflect a wider role for Pol δ in any situation where the 3′ end of the leading strand becomes uncoupled from the advancing replication fork. Our data suggest uncoupling is prevented during unperturbed replication by connection of the 3′ end of the leading strand to the CMG helicase by PCNA-Pol ε interactions. Nonetheless, uncoupling may occur under conditions of replication stress, for example, when leading-strand synthesis is blocked by DNA damage in the template. We propose that, once the damage is repaired, Pol δ will have a critical role in re-establishing coupled leading-strand synthesis by temporarily taking over rapid leading-strand synthesis until

the 3′ end of the leading strand is reconnected with the advancing CMG. We speculate that high levels of nucleotide misincorporation without rapid repair may, like DNA damage, promote uncoupling and increase the contribution of Pol δ in leading-strand replication, which may partly explain the observation that Pol δ appears to play a significant role in leading-strand synthesis when certain mismatch repair mutants are combined with DNA polymerase proofreading mutants (Johnson et al., 2015).

The eukaryotic replisome must coordinate replication with many nuclear processes, including sister chromatid cohesion, telomere replication, epigenetic inheritance of gene expression patterns, and post-replication repair. In addition, the replisome must deal with obstacles, including DNA damage, nucleosomes, and transcription complexes from all three nuclear RNA polymerases. The availability of the reconstituted replisome opens new avenues for understanding these interactions.

EXPERIMENTAL PROCEDURES

Details of protein purification, template construction, and data analysis are provided in the Supplemental Experimental Procedures.

Soluble Replication Reactions

All steps were conducted at 30°C. MCM loading (5–10 μL per lane or 50–100 μL for time course experiments) was conducted in a buffer containing 25 mM HEPES-KOH (pH 7.6), 100 mM potassium glutamate, 10 mM magnesium acetate, 100 μg/mL BSA, 1 mM DTT, 0.01% NP-40-S, 5 mM ATP, 45 nM Cdc6, 22.5 nM ORC, 100 nM Cdt1-Mcm2-7, and 4 nM circular DNA template. Reactions were incubated for 20 min, at which point DDK was added to 25 nM and incubation was continued for a further 20 min. The reaction volume was then increased 2-fold by the addition of pre-equilibrated buffer to give a final replication reaction buffer of 25 mM HEPES-KOH (pH 7.6); 100–250 mM potassium glutamate (see figure legends for details); 10 mM magnesium acetate; 100 μg/mL BSA; 1 mM DTT; 0.01% NP40-S; 3 mM ATP; 22.5 nM Cdc6; 11.3 nM ORC; 50 nM Cdt1-Mcm2-7; 12.5 nM DDK; 2 nM circular DNA template; 200 μM CTP, GTP, and UTP; 80 μM dCTP, dGTP, dATP, and dTTP; and 33 nM α^{32}P-dCTP. Replication was initiated by adding a master mix of proteins to give final concentrations (unless stated otherwise in the figure legends) of 25 nM Sld3/7, 50 nM Sld2, 30 nM Dpb11, 210 nM GINS, 40 nM Cdc45, 20 nM Pol ε, 5 nM Mcm10, 20 nM Ctf4, 100 nM RPA, 20 nM S-CDK, 20 nM Pol α, 20 nM Csm3/Tof1, 10–20 nM Mrc1, 20 nM RFC, 10–20 nM PCNA, 10 nM Topo I, 20 nM Topo II, and 10 nM Pol δ. The volume of proteins added to initiate replication typically constituted 15% of the final reaction volume and contributed ~18.5 mM KCl/NaCl, ~18 mM KOAc, and 2.5% glycerol. Following incubation (see figure legends for reaction times), reactions were quenched by the addition of an equal volume of 50 mM EDTA. Unincorporated nucleotide was removed with illusta MicroSpin G-50 columns (GE Healthcare), and samples were separated through 0.6% alkaline agarose gels as described (Yeeles et al., 2015).

Pulse-Chase Experiments

Pulse-chase experiments were performed using the same conditions as for soluble reactions, except that 40 nM Pol α was used in all experiments and the concentration of dCTP in the pulse was reduced to 4 μM for the experiments in Figures 2C, 4B, 5E and Figure S6C and 2 μM for Figures 6F, 7A and Figures S7A and S7C. The concentrations of dCTP, dGTP, dATP, and dTTP were then increased to 600 μM during the chase.

Primer Extension Reactions

Primer extension reactions were conducted in the standard replication buffer excluding the proteins required for origin firing, Ctf4, Topo I, and Topo II. Reactions contained 1 nM primed M13mp18 ssDNA that was incubated for 5 min at 30°C with 20 nM PCNA, 20 nM RFC, and 400 nM RPA. Replication was then initiated by the addition of Pol δ to 10 nM. Aliquots were withdrawn and were processed as described for soluble replication reactions.

SUPPLEMENTAL INFORMATION

Supplemental Information includes Supplemental Experimental Procedures, seven figures, and four tables and can be found with this article online at http://dx.doi.org/10.1016/j.molcel.2016.11.017.

AUTHOR CONTRIBUTIONS

J.T.P.Y. performed all the experiments. A.J. provided Pol ε proteins. A.E. helped design and construct overexpression strains. J.T.P.Y. and J.F.X.D. designed the experiments and wrote the paper.

ACKNOWLEDGMENTS

We thank C. Kurat for FACT protein and G. Coster for advice on origin sequences. We thank A. Alidoust and N. Patel for growing yeast cultures. This work was supported by the Francis Crick Institute, which receives its core funding from Cancer Research UK (FC001066), the UK Medical Research Council (FC001066), and the Wellcome Trust (FC001066). It also was funded by a Wellcome Senior Investigator Award (106252/Z/14/Z) and an ERC Advanced Grant (669424-CHROMOREP) to J.F.X.D., an FEBS Return-to-Europe fellowship to J.T.P.Y., and a Boehringer Ingelheim Fonds PhD fellowship to A.J.

Received: July 18, 2016
Revised: October 17, 2016
Accepted: November 7, 2016
Published: December 15, 2016

REFERENCES

Bando, M., Katou, Y., Komata, M., Tanaka, H., Itoh, T., Sutani, T., and Shirahige, K. (2009). Csm3, Tof1, and Mrc1 form a heterotrimeric mediator complex that associates with DNA replication forks. J. Biol. Chem. *284*, 34355–34365.

Calzada, A., Hodgson, B., Kanemaki, M., Bueno, A., and Labib, K. (2005). Molecular anatomy and regulation of a stable replisome at a paused eukaryotic DNA replication fork. Genes Dev. *19*, 1905–1919.

Chilkova, O., Stenlund, P., Isoz, I., Stith, C.M., Grabowski, P., Lundström, E.B., Burgers, P.M., and Johansson, E. (2007). The eukaryotic leading and lagging strand DNA polymerases are loaded onto primer-ends via separate mechanisms but have comparable processivity in the presence of PCNA. Nucleic Acids Res. *35*, 6588–6597.

Clausen, A.R., Lujan, S.A., Burkholder, A.B., Orebaugh, C.D., Williams, J.S., Clausen, M.F., Malc, E.P., Mieczkowski, P.A., Fargo, D.C., Smith, D.J., and Kunkel, T.A. (2015). Tracking replication enzymology in vivo by genome-wide mapping of ribonucleotide incorporation. Nat. Struct. Mol. Biol. *22*, 185–191.

Conti, C., Saccà, B., Herrick, J., Lalou, C., Pommier, Y., and Bensimon, A. (2007). Replication fork velocities at adjacent replication origins are coordinately modified during DNA replication in human cells. Mol. Biol. Cell *18*, 3059–3067.

Daigaku, Y., Keszthelyi, A., Müller, C.A., Miyabe, I., Brooks, T., Retkute, R., Hubank, M., Nieduszynski, C.A., and Carr, A.M. (2015). A global profile of replicative polymerase usage. Nat. Struct. Mol. Biol. *22*, 192–198.

Dalgaard, J.Z., and Klar, A.J. (2000). swi1 and swi3 perform imprinting, pausing, and termination of DNA replication in S. pombe. Cell *102*, 745–751.

Deegan, T.D., Yeeles, J.T., and Diffley, J.F. (2016). Phosphopeptide binding by Sld3 links Dbf4-dependent kinase to MCM replicative helicase activation. EMBO J. *35*, 961–973.

Douglas, M.E., and Diffley, J.F.X. (2016). Recruitment of Mcm10 to sites of replication initiation requires direct binding to the Minichromosome Maintenance (MCM) complex. J. Biol. Chem. *291*, 5879–5888.

Dua, R., Levy, D.L., Li, C.M., Snow, P.M., and Campbell, J.L. (2002). In vivo reconstitution of Saccharomyces cerevisiae DNA polymerase epsilon in insect cells. Purification and characterization. J. Biol. Chem. *277*, 7889–7896.

Evrin, C., Clarke, P., Zech, J., Lurz, R., Sun, J., Uhle, S., Li, H., Stillman, B., and Speck, C. (2009). A double-hexameric MCM2-7 complex is loaded onto origin DNA during licensing of eukaryotic DNA replication. Proc. Natl. Acad. Sci. USA *106*, 20240–20245.

Gambus, A., Jones, R.C., Sanchez-Diaz, A., Kanemaki, M., van Deursen, F., Edmondson, R.D., and Labib, K. (2006). GINS maintains association of Cdc45 with MCM in replisome progression complexes at eukaryotic DNA replication forks. Nat. Cell Biol. *8*, 358–366.

Gambus, A., van Deursen, F., Polychronopoulos, D., Foltman, M., Jones, R.C., Edmondson, R.D., Calzada, A., and Labib, K. (2009). A key role for Ctf4 in coupling the MCM2-7 helicase to DNA polymerase alpha within the eukaryotic replisome. EMBO J. *28*, 2992–3004.

Georgescu, R.E., Langston, L., Yao, N.Y., Yurieva, O., Zhang, D., Finkelstein, J., Agarwal, T., and O'Donnell, M.E. (2014). Mechanism of asymmetric polymerase assembly at the eukaryotic replication fork. Nat. Struct. Mol. Biol. *21*, 664–670.

Georgescu, R.E., Schauer, G.D., Yao, N.Y., Langston, L.D., Yurieva, O., Zhang, D., Finkelstein, J., and O'Donnell, M.E. (2015). Reconstitution of a eukaryotic replisome reveals suppression mechanisms that define leading/lagging strand operation. eLife *4*, e04988.

Hodgson, B., Calzada, A., and Labib, K. (2007). Mrc1 and Tof1 regulate DNA replication forks in different ways during normal S phase. Mol. Biol. Cell *18*, 3894–3902.

Johnson, R.E., Klassen, R., Prakash, L., and Prakash, S. (2015). A major role of DNA polymerase δ in replication of both the leading and lagging DNA strands. Mol. Cell *59*, 163–175.

Kanemaki, M., and Labib, K. (2006). Distinct roles for Sld3 and GINS during establishment and progression of eukaryotic DNA replication forks. EMBO J. *25*, 1753–1763.

Katou, Y., Kanoh, Y., Bando, M., Noguchi, H., Tanaka, H., Ashikari, T., Sugimoto, K., and Shirahige, K. (2003). S-phase checkpoint proteins Tof1 and Mrc1 form a stable replication-pausing complex. Nature *424*, 1078–1083.

Kesti, T., Flick, K., Keränen, S., Syväoja, J.E., and Wittenberg, C. (1999). DNA polymerase epsilon catalytic domains are dispensable for DNA replication, DNA repair, and cell viability. Mol. Cell *3*, 679–685.

Komata, M., Bando, M., Araki, H., and Shirahige, K. (2009). The direct binding of Mrc1, a checkpoint mediator, to Mcm6, a replication helicase, is essential for the replication checkpoint against methyl methanesulfonate-induced stress. Mol. Cell. Biol. *29*, 5008–5019.

Krings, G., and Bastia, D. (2004). swi1- and swi3-dependent and independent replication fork arrest at the ribosomal DNA of Schizosaccharomyces pombe. Proc. Natl. Acad. Sci. USA *101*, 14085–14090.

Kurat, C.F., Yeeles, J.T.P., Patel, H., Early, A., and Diffley, J.F.X. (2016). Chromatin Controls DNA Replication Origin Selection, Lagging-Strand Synthesis, and Replication Fork Rates. Mol. Cell *65*, this issue, 117–130.

Langston, L.D., Zhang, D., Yurieva, O., Georgescu, R.E., Finkelstein, J., Yao, N.Y., Indiani, C., and O'Donnell, M.E. (2014). CMG helicase and DNA polymerase ε form a functional 15-subunit holoenzyme for eukaryotic leading-strand DNA replication. Proc. Natl. Acad. Sci. USA *111*, 15390–15395.

Liachko, I., Youngblood, R.A., Keich, U., and Dunham, M.J. (2013). High-resolution mapping, characterization, and optimization of autonomously replicating sequences in yeast. Genome Res. *23*, 698–704.

Lou, H., Komata, M., Katou, Y., Guan, Z., Reis, C.C., Budd, M., Shirahige, K., and Campbell, J.L. (2008). Mrc1 and DNA polymerase epsilon function together in linking DNA replication and the S phase checkpoint. Mol. Cell *32*, 106–117.

Mohanty, B.K., Bairwa, N.K., and Bastia, D. (2006). The Tof1p-Csm3p protein complex counteracts the Rrm3p helicase to control replication termination of Saccharomyces cerevisiae. Proc. Natl. Acad. Sci. USA *103*, 897–902.

Moyer, S.E., Lewis, P.W., and Botchan, M.R. (2006). Isolation of the Cdc45/Mcm2-7/GINS (CMG) complex, a candidate for the eukaryotic DNA replication fork helicase. Proc. Natl. Acad. Sci. USA *103*, 10236–10241.

Nick McElhinny, S.A., Gordenin, D.A., Stith, C.M., Burgers, P.M., and Kunkel, T.A. (2008). Division of labor at the eukaryotic replication fork. Mol. Cell *30*, 137–144.

Pacek, M., Tutter, A.V., Kubota, Y., Takisawa, H., and Walter, J.C. (2006). Localization of MCM2-7, Cdc45, and GINS to the site of DNA unwinding during eukaryotic DNA replication. Mol. Cell *21*, 581–587.

Petermann, E., Helleday, T., and Caldecott, K.W. (2008). Claspin promotes normal replication fork rates in human cells. Mol. Biol. Cell *19*, 2373–2378.

Prelich, G., and Stillman, B. (1988). Coordinated leading and lagging strand synthesis during SV40 DNA replication in vitro requires PCNA. Cell *53*, 117–126.

Quan, Y., Xia, Y., Liu, L., Cui, J., Li, Z., Cao, Q., Chen, X.S., Campbell, J.L., and Lou, H. (2015). Cell-cycle-regulated interaction between Mcm10 and double hexameric Mcm2-7 is required for helicase splitting and activation during S phase. Cell Rep. *13*, 2576–2586.

Remus, D., Beuron, F., Tolun, G., Griffith, J.D., Morris, E.P., and Diffley, J.F.X. (2009). Concerted loading of Mcm2-7 double hexamers around DNA during DNA replication origin licensing. Cell *139*, 719–730.

Ricke, R.M., and Bielinsky, A.K. (2004). Mcm10 regulates the stability and chromatin association of DNA polymerase-alpha. Mol. Cell *16*, 173–185.

Sekedat, M.D., Fenyö, D., Rogers, R.S., Tackett, A.J., Aitchison, J.D., and Chait, B.T. (2010). GINS motion reveals replication fork progression is remarkably uniform throughout the yeast genome. Mol. Syst. Biol. *6*, 353.

Sengupta, S., van Deursen, F., de Piccoli, G., and Labib, K. (2013). Dpb2 integrates the leading-strand DNA polymerase into the eukaryotic replisome. Curr. Biol. *23*, 543–552.

Smith, D.J., and Whitehouse, I. (2012). Intrinsic coupling of lagging-strand synthesis to chromatin assembly. Nature *483*, 434–438.

Szyjka, S.J., Viggiani, C.J., and Aparicio, O.M. (2005). Mrc1 is required for normal progression of replication forks throughout chromatin in S. cerevisiae. Mol. Cell *19*, 691–697.

Tanaka, S., and Araki, H. (2013). Helicase activation and establishment of replication forks at chromosomal origins of replication. Cold Spring Harb. Perspect. Biol. *5*, a010371.

Tanaka, S., Umemori, T., Hirai, K., Muramatsu, S., Kamimura, Y., and Araki, H. (2007). CDK-dependent phosphorylation of Sld2 and Sld3 initiates DNA replication in budding yeast. Nature *445*, 328–332.

Tanaka, H., Katou, Y., Yagura, M., Saitoh, K., Itoh, T., Araki, H., Bando, M., and Shirahige, K. (2009). Ctf4 coordinates the progression of helicase and DNA polymerase alpha. Genes Cells *14*, 807–820.

Tourrière, H., Versini, G., Cordón-Preciado, V., Alabert, C., and Pasero, P. (2005). Mrc1 and Tof1 promote replication fork progression and recovery independently of Rad53. Mol. Cell *19*, 699–706.

van Deursen, F., Sengupta, S., De Piccoli, G., Sanchez-Diaz, A., and Labib, K. (2012). Mcm10 associates with the loaded DNA helicase at replication origins and defines a novel step in its activation. EMBO J. *31*, 2195–2206.

Yeeles, J.T., Deegan, T.D., Janska, A., Early, A., and Diffley, J.F.X. (2015). Regulated eukaryotic DNA replication origin firing with purified proteins. Nature *519*, 431–435.

Yu, C., Gan, H., Han, J., Zhou, Z.X., Jia, S., Chabes, A., Farrugia, G., Ordog, T., and Zhang, Z. (2014). Strand-specific analysis shows protein binding at replication forks and PCNA unloading from lagging strands when forks stall. Mol. Cell *56*, 551–563.

Zegerman, P., and Diffley, J.F.X. (2007). Phosphorylation of Sld2 and Sld3 by cyclin-dependent kinases promotes DNA replication in budding yeast. Nature *445*, 281–285.

Molecular Cell, Volume *65*

Supplemental Information

How the Eukaryotic Replisome Achieves Rapid and Efficient DNA Replication

Joseph T.P. Yeeles, Agnieska Janska, Anne Early, and John F.X. Diffley

Figure S1

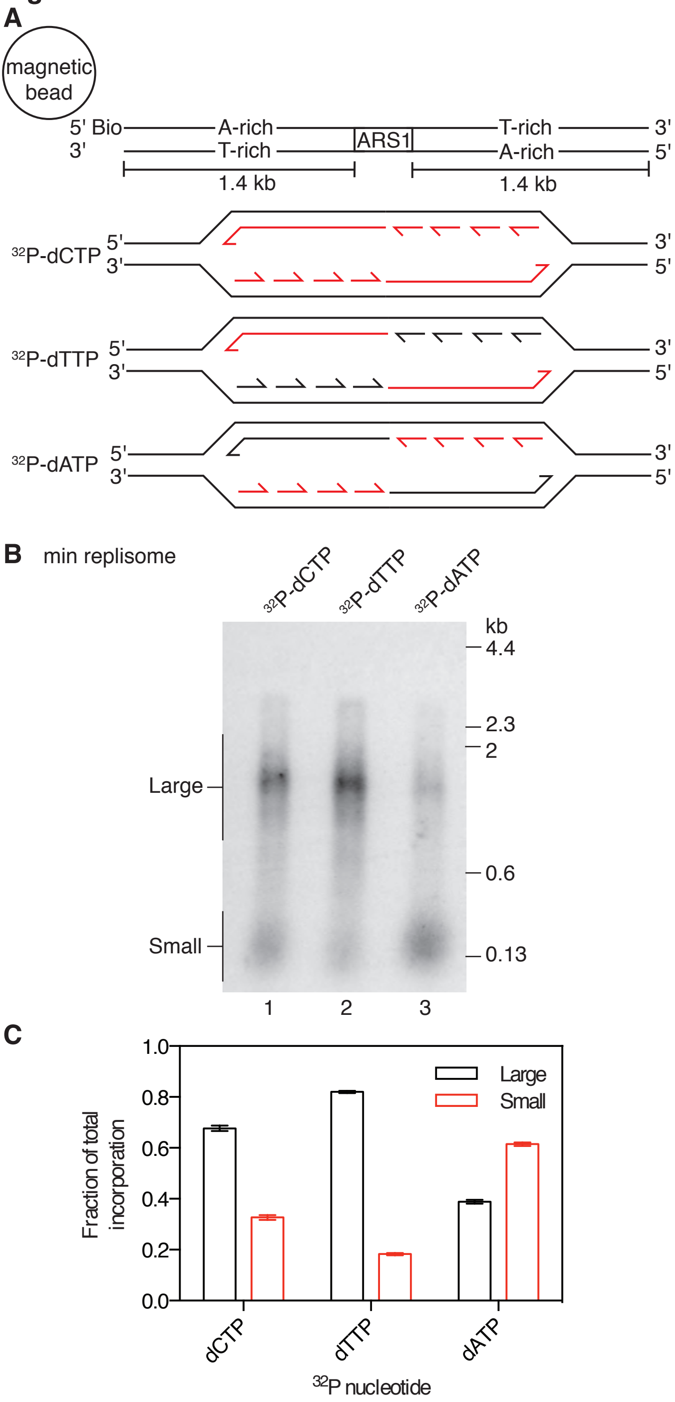
A
magnetic bead
5' Bio
A-rich
T-rich
ARS1
3'
5'
1.4 kb
1.4 kb
32P-dCTP
32P-dTTP
32P-dATP
B
min replisome
kb
4.4
2.3
2
0.6
0.13
Large
Small
1
2
3
C
Fraction of total incorporation
1.0
0.8
0.6
0.4
0.2
0.0
Large
Small
dCTP
dTTP
dATP
32P nucleotide

Figure S2

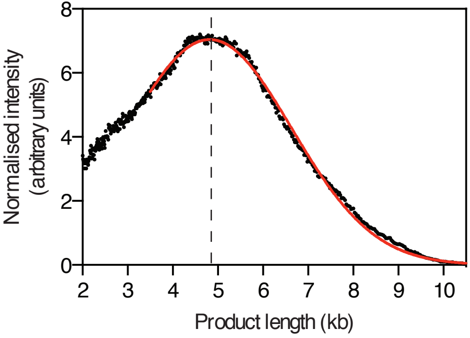
8
6
4
2
0
Normalised intensity
(arbitrary units)
2
3
4
5
6
7
8
9
10
Product length (kb)

Figure S3

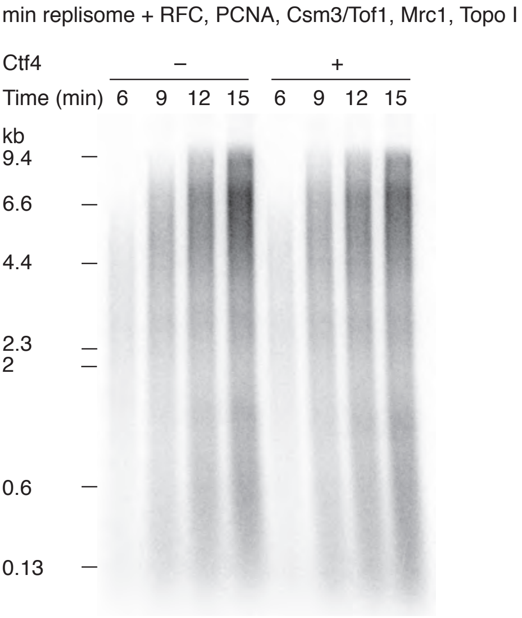

min replisome + RFC, PCNA, Csm3/Tof1, Mrc1, Topo I
Ctf4
–
+
Time (min)
6
9
12
15
6
9
12
15
kb
9.4
6.6
4.4
2.3
2
0.6
0.13

Figure S4

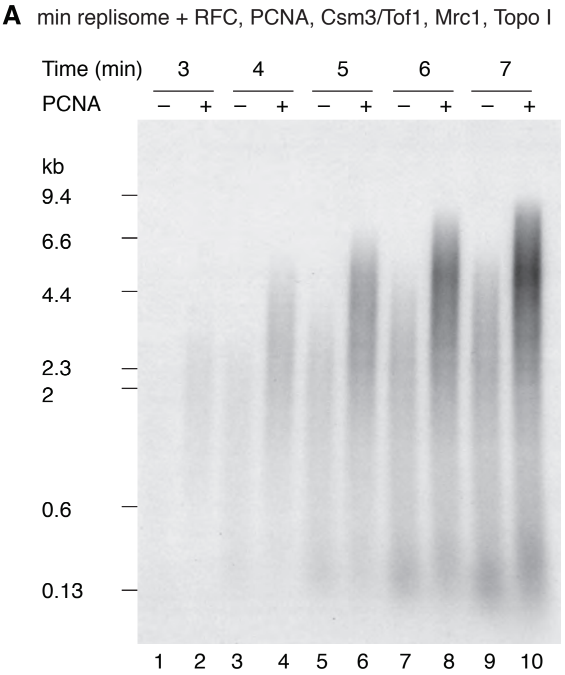

A
min replisome + RFC, PCNA, Csm3/Tof1, Mrc1, Topo I
Time (min) 3 4 5 6 7
PCNA – + – + – + – + – +
kb
9.4
6.6
4.4
2.3
2
0.6
0.13
1 2 3 4 5 6 7 8 9 10

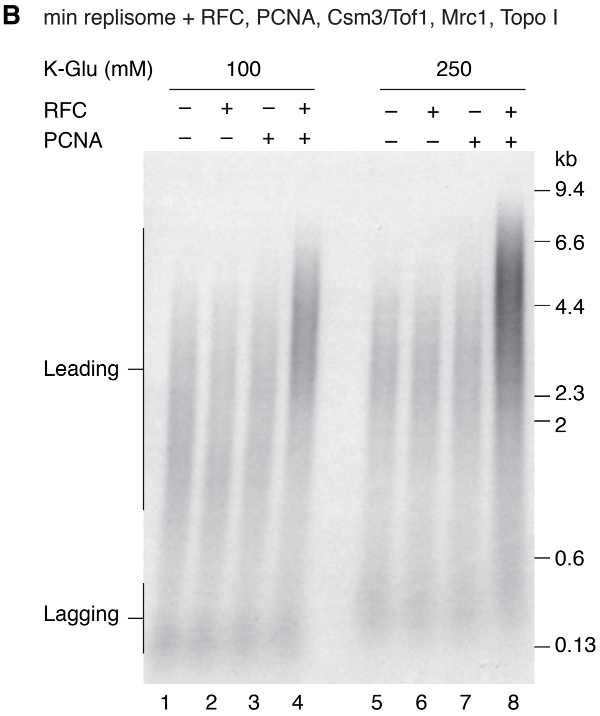

B
min replisome + RFC, PCNA, Csm3/Tof1, Mrc1, Topo I
K-Glu (mM) 100 250
RFC – + – + – + – +
PCNA – – + + – – + +
kb
9.4
6.6
4.4
2.3
2
0.6
0.13
Leading
Lagging
1 2 3 4 5 6 7 8

Figure S5

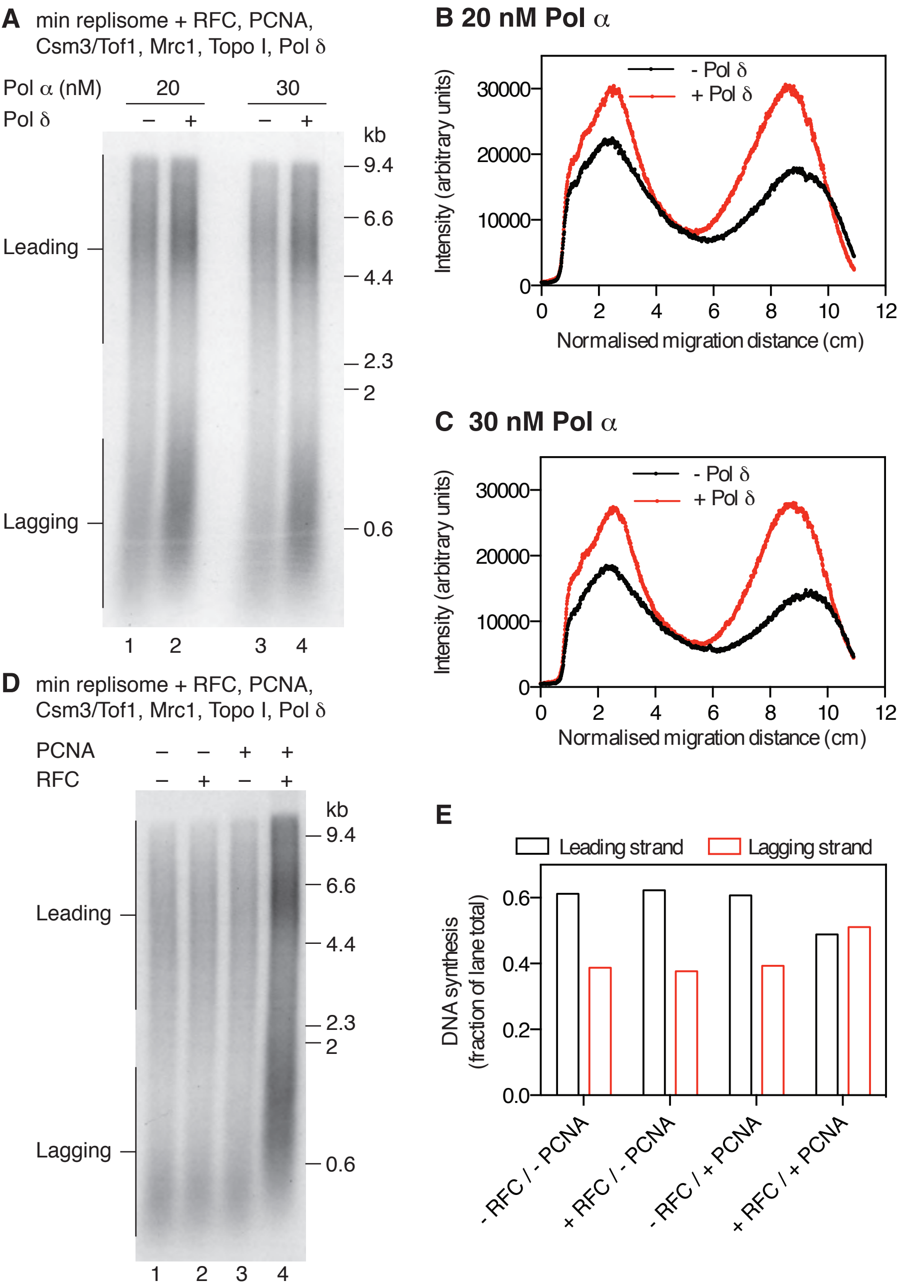

A
min replisome + RFC, PCNA, Csm3/Tof1, Mrc1, Topo I, Pol δ
Pol α (nM)
20
30
Pol δ
–
+
–
+
kb
9.4
6.6
4.4
2.3
2
0.6
Leading
Lagging
1
2
3
4
B 20 nM Pol α
- Pol δ
+ Pol δ
Intensity (arbitrary units)
Normalised migration distance (cm)
C 30 nM Pol α
- Pol δ
+ Pol δ
Intensity (arbitrary units)
Normalised migration distance (cm)
D
min replisome + RFC, PCNA, Csm3/Tof1, Mrc1, Topo I, Pol δ
PCNA
RFC
kb
9.4
6.6
4.4
2.3
2
0.6
Leading
Lagging
1
2
3
4
E
Leading strand
Lagging strand
DNA synthesis (fraction of lane total)
- RFC / - PCNA
+ RFC / - PCNA
- RFC / + PCNA
+ RFC / + PCNA

Figure S6

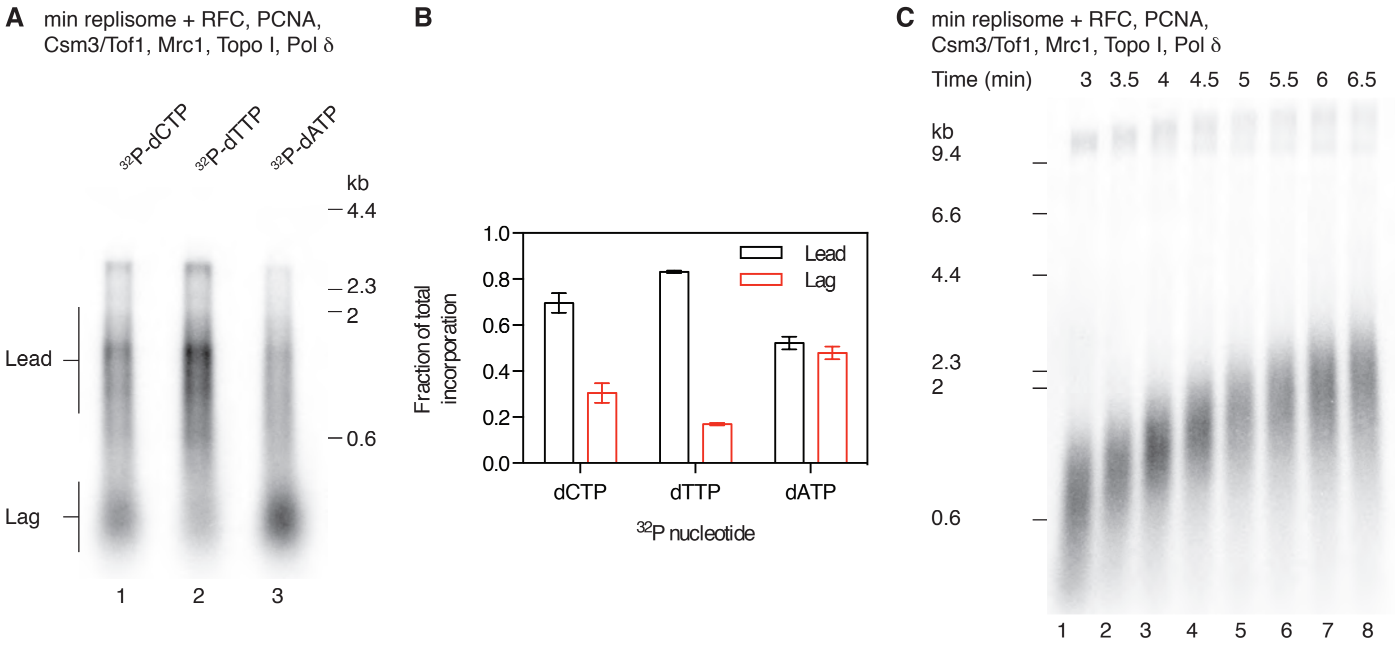

A
min replisome + RFC, PCNA, Csm3/Tof1, Mrc1, Topo I, Pol δ
32P-dCTP
32P-dTTP
32P-dATP
kb
4.4
2.3
2
0.6
Lead
Lag
1
2
3
B
1.0
0.8
0.6
0.4
0.2
0.0
Fraction of total incorporation
Lead
Lag
dCTP
dTTP
dATP
32P nucleotide
C
min replisome + RFC, PCNA, Csm3/Tof1, Mrc1, Topo I, Pol δ
Time (min)
3
3.5
4
4.5
5
5.5
6
6.5
kb
9.4
6.6
4.4
2.3
2
0.6
1
2
3
4
5
6
7
8

Figure S7

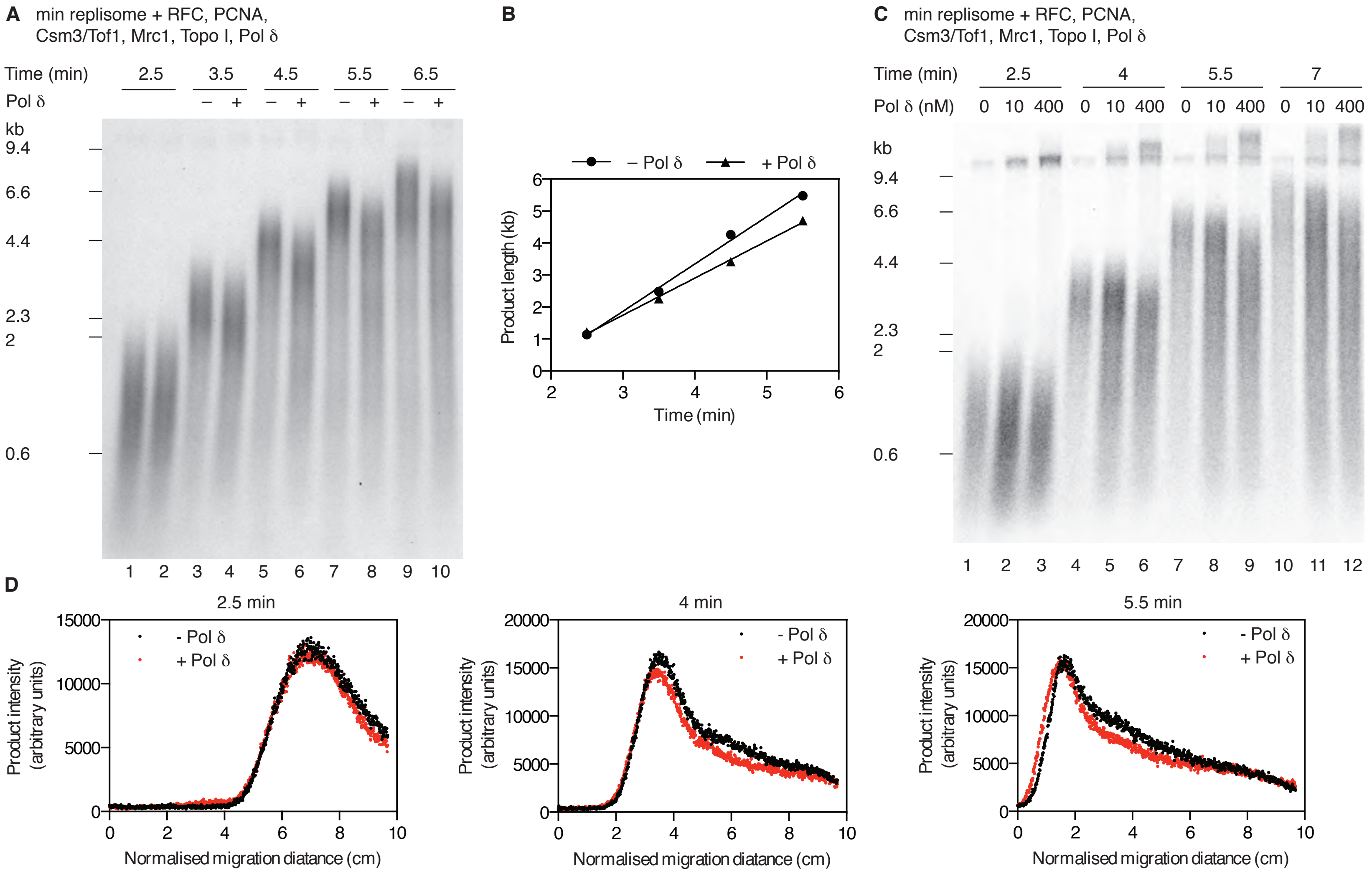

A min replisome + RFC, PCNA, Csm3/Tof1, Mrc1, Topo I, Pol δ
Time (min) 2.5 3.5 4.5 5.5 6.5
Pol δ − + − + − + − +
kb 9.4 6.6 4.4 2.3 2 0.6
1 2 3 4 5 6 7 8 9 10
B
− Pol δ + Pol δ
Product length (kb)
Time (min)
C min replisome + RFC, PCNA, Csm3/Tof1, Mrc1, Topo I, Pol δ
Time (min) 2.5 4 5.5 7
Pol δ (nM) 0 10 400 0 10 400 0 10 400 0 10 400
kb 9.4 6.6 4.4 2.3 2 0.6
1 2 3 4 5 6 7 8 9 10 11 12
D
2.5 min
4 min
5.5 min
- Pol δ
+ Pol δ
Product intensity (arbitrary units)
Normalised migration diatance (cm)

Supplemental Figure Legends

Figure S1. Related to Figure 1.

(A) Schematic representation of the template used to assign leading and lagging strand replication products. The template contains a functional 100 bp fragment of ARS1 at the centre (Liachko et al., 2013). Labelled nascent replication products are drawn in red. [α-^{32}P] dCTP will label nascent leading and lagging strands evenly. [α-^{32}P] dTTP will preferentially label nascent leading strands. Lagging strands will be preferentially labelled with [α-^{32}P] dATP.

(B) Bead-coupled replication reactions performed under origin-specific conditions. Reactions were performed for 20 min. Topo II was not present in the reaction.

(C) Quantitation of small and large products from reactions performed as described in (B). Data were normalised to the sum of small and large products for each labelled nucleotide. Data represent the average of four independent experiments and error bars show the standard error of the mean (SEM).

Figure S2. Related to Figure 1.

Normalised lane profile showing leading strand products for the 120 min time point in Figure 1B. Because products are continuously labelled in replication reactions, signal intensity is proportional to product length. To derive an unbiased distribution of replication products, signal intensity was divided by product length. Data were fit to a gaussian distribution. Products below 3.5 kb were excluded from the fit to avoided interference from the population of lagging strand products. The dotted vertical line shows the mean of the distribution.

Figure S3. Related to Figure 3.

Replication time course performed as in Figure 3A in the presence or absence of Ctf4.

Figure S4. Related to Figure 4.

(A) Time course performed as in Figure 4A but with 40 nM Pol α.

(B) Experiments performed as in Figure 4A for 6 minutes at 100 mM and 250 mM potassium glutamate. PCNA and RFC were included where indicated.

Figure S5. Related to Figure 5.

(A) Experiment conducted as in Figure 5A for 20 min.

(B and C) Lane profiles for the data in (A).

(D) Replication reaction conducted as in (A) for 15 min but with Pol δ present in all lanes.

(E) Quantitation of the leading and lagging strand products in (D).

Figure S6. Related to Figure 6.

(A) Bead coupled replication reaction performed as in Figure S1A with the following modifications. Pol ε- Δ cat was used in place of Pol ε, reactions contained 200 mM potassium glutamate and 5 nM Pol δ, and replication was performed for 30 min.

(B) Quantitation of experiments performed as described in A. Data represent the mean of 3 repeats and error bars show the SEM.

(C) Pulse chase reaction performed as in Figure 2C but with Pol ε-Δcat in place of Pol ε, and 10 nM Pol δ. The chase was added at 2 min 50 s.

Figure S7. Related to Figure 7.

(A) Pulse chase experiment performed as in Figure 6F. 400 nM Pol δ was included in the chase where indicated.

(B) Quantitation of the data in (A). Data represent the peaks of the distributions of leading strand products.

(C) Pulse chase reaction essentially as performed in (A), except that Pol δ was included from the beginning of the reactions where indicated.

(D) Lane scans of data in Figure 7A where Pol δ was absent in the pulse, and was then either present or absent during the chase.

Supplemental Tables

Supplemental table 1 | Affinity tag strategies for protein purification, related to Experimental Procedures

Protein	Yeast strain	Affinity tag strategy	Affinity tag sequence
Mrc1	yJY32	C-terminal 2xFLAG	DYKDDDGDYKDDD
Pol δ	yAE34	C-terminal TEV-CBP tag on Pol32	ENLYFQGEKRRWKKNFIAVSAANRFKKISSS GAL
RFC	yAE41	N-terminal TEV-CBP tag on RFC3	MKRRWKKNFIAVSAANRFKKISSSGALENLYF QGE
Topo I	yAE42	N-terminal TEV-CBP tag	MKRRWKKNFIAVSAANRFKKISSSGALENLYF QGE
PCNA	yAE43	N-terminal TEV-CBP tag	MKRRWKKNFIAVSAANRFKKISSSGALENLYF QGE
Csm3/Tof1	yAE48	N-terminal TEV-CBP tag on Csm3	MKRRWKKNFIAVSAANRFKKISSSGALENLYF QGE
Pol ε – Δcat	yAJ25	C-terminal TEV-CBP tag on Dpb4	ENLYFQGEKRRWKKNFIAVSAANRFKKISSS GAL

All N-terminal tags are located immediately upstream of the original start codon
All C-terminal tags are located immediately before the original stop

Supplemental table 2 | *Saccharomyces cerevisiae* strains, related to Experimental Procedures

Strain	Genotype	Reference
yAE34	*MATa ade2-1 ura3-1 his3-11,15 trp1-1 leu2-3,112 can1-100*<br>*bar1::Hyg*<br>*pep4::KanMX*<br>*ura3::URA3pRS306-POL31+POL3*<br>*his3::HIS3pRS303-Pol32-CBP+Gal4*	This study
yAE41	*MATa ade2-1 ura3-1 his3-11,15 trp1-1 leu2-3,112 can1-100*<br>*bar1::Hyg*<br>*pep4::KanMX*<br>*ura3::URA3pRS306-RFC2+CBP-RFC3*<br>*trp1::TRP1pRS304-RFC4 + RFC5*<br>*his3::HIS3pRS303-RFC1+Gal4*	This study
yAE42	*MATa ade2-1 ura3-1 his3-11,15 trp1-1 leu2-3,112 can1-100*<br>*bar1::Hyg*<br>*pep4::KanMX*<br>*his3::HIS3pRS303-CBP-TOP1+Gal4*	This study
yAE43	*MATa ade2-1 ura3-1 his3-11,15 trp1-1 leu2-3,112 can1-100*<br>*bar1::Hyg*<br>*pep4::KanMX*<br>*his3::HIS3pRS303-CBP-Pol30+Gal4*	This study
yAE48	*MATa ade2-1 ura3-1 his3-11,15 trp1-1 leu2-3,112 can1-100*<br>*bar1::Hyg*<br>*pep4::KanMX*<br>*ura3::URA3pRS306-CBP-Csm3 + Tof1*	This study
yJY32	*MATa ade2-1 ura3-1 his3-11,15 trp1-1 leu2-3,112 can1-100*<br>*bar1::Hyg*<br>*pep4::KanMX*<br>*his3::HIS3pRS303-Mrc1 C-term 2x Flag*	This study
yAJ25	*MATa ade2-1 ura3-1 his3-11,15 trp1-1 leu2-3,112 can1-100*<br>*bar1::Hyg*<br>*pep4::KanMX*<br>*ura3::URA3pRS306/Dpb2, Dpb3*<br>*trp1::TRP1pRS304/ Pol2Δ1262, Dpb4-Tev-CBP*	This study

Supplemental table 3 | Plasmids, related to Experimental Procedures

Plasmid	Original vector*	Insert	Plasmid construction
vJY17	pJF2	Mrc1-2x Flag	Synthetic construct cloned 5′ - SgrAI , 3′ - NotI
pRS306/Pol3-Gal-Pol31	pJF5	Pol3<br>Pol31	Synthetic construct cloned 5′- SgrAI , 3′- NotI<br>Synthetic construct cloned 5′- AscI , 3′- Xho1
pRS303/Pol32-CBP-Gal-Gal4	pJF2	Pol32-CBP	Synthetic construct cloned 5′- SgrAI , 3′- NotI
pRS306/RFC2-Gal-CBP-RFC3	pJF5	RFC2<br>CBP-RFC3	Synthetic construct cloned 5′- SgrAI , 3′- NotI<br>Synthetic construct cloned 5′- AscI , 3′- Xho1
pRS305/RFC5-Gal-RFC4	pJF3	RFC5<br>RFC4	Synthetic construct cloned 5′- SgrAI , 3′- NotI<br>Synthetic construct cloned 5′- AscI , 3′- Xho1
pRS303/RFC1-Gal-Gal4	pJF2	RFC1	Synthetic construct cloned 5′- SgrAI , 3′- NotI
pRS303/CBP-Pol30-Gal-Gal4	pJF2	Pol30	Synthetic construct cloned 5′- SgrAI , 3′- NotI
pRS303/CBP-Top1-Gal-Gal4	pJF2	CBP-Top1	Synthetic construct cloned 5′- SgrAI , 3′- NotI
pRS306/Tof1-Gal-CBP-Csm3	pJF5	Tof1<br>CBP-Csm3	Synthetic construct cloned 5′- SgrAI , 3′- NotI<br>Synthetic construct cloned 5′- AscI , 3′- Xho1
vJY19	pET28a	PCNA	Native PCNA gene PCR amplified from W303 genomic DNA with oligos JTY149 and JTY150. PCR product was digested with Pci1 and BamH1 and ligated into pET28a linearised with Nde1 and BamH1.
pRS306 (Dpb2 + Dpb3)	pJF19	Dpb2<br>Dpb3	Synthetic construct cloned 5′- SgrAI , 3′- NotI<br>Synthetic construct cloned 5′- AscI , 3′- Xho1
pRS304 (Δ 1262 + Dpb4-CBP)	pRS304 (Pol2 + Dpb4-CBP)	Pol2Δ1262<br>Dpb4-CBP	Synthetic construct cloned 5′- AscI , 3′- Xho1<br>Synthetic construct cloned 5′- SgrAI , 3′- NotI

Synthetic constructs were codon optimised for expression in *Saccharomyces cerevisiae* (Sharp and Li, 1987). Sequences from the 5′ and 3′ end of the PGK1 gene were added to all synthetic genes except Mrc1. Genes were synthesised by GeneArt Gene Synthesis (Life technologies).

* For details of the original expression vectors see (Coster et al., 2014; Frigola et al., 2013).

Supplemental table 4 | Oligonucleotides, related to Experimental Procedures

Oligonucleotide	Sequence
JTY107	TTCTAGAAAACCCGGGCTTTGATATGCCGGAAGGAGTTG
JTY108	TCCACTGGGTGGTACCGTGTTAGCACAATCCATATGCC
JTY161	Bio-CCGTGCCAAAGAAGAAAGTC
JTY164	GCAGGTCGACTCTAGATACGAAC
JTY149	GTGTATAAAAACATGTTGGAAGCTAAGTTCGAAGAAGC
JTY150	TTCAATTCAAGGATCCTTATTCTTCATCGTTGAACTTTGG
JTY180	GAATAATGGAAGGGTTAGAACCTACCAT
AJ34	ATAAATGGCGCGCCATGCCATCTATGGACGAAGACTA
AJ32	ATTATACTCGAGGCATAAAGGCATTAAAAGAG

Supplemental Experimental Procedures

Replication templates

An approximately 7.7 kb region surrounding ARS1 was amplified from *S. cerevisiae* W303 genomic DNA by PCR with primers JTY107 and JTY108. The PCR product was then cloned into pBluescript KS+ using the restriction enzymes Kpn I and Xma I to give the plasmid vJY22. The sequence of ARS1 was confirmed by sequencing. For soluble replication reactions covalently closed plasmid templates were purified using a Qiagen Maxi Prep Kit followed by one round of caesium chloride density gradient centrifugation.

Primed M13mp18 ssDNA was prepared by annealing the oligonucleotide JTY180 to M13mp18 single stranded DNA (New England Biolabs). Excess oligonucleotide was removed using an S-400 column (GE Healthcare).

The linear bead-coupled DNA templates used to differentiate leading and lagging strand products (Figures S1 and S6) were generated by PCR using the primers JTY161 and JTY164 with the synthetic DNA construct JY_String_1

(GeneArt) as template. DNA was coupled to Dynabeads M-280 Streptavidin (Invitrogen) as described previously (Yeeles et al., 2015).

JY_String_1

CCGTGCCAAAGAAGAAAGTCAATGACCCGTACGCCACGTACGAAACCGGTGAATCAATG
CCACACAGAGAGAAGGGAGTGGGTAAACACAGAACCCGGCGGCCAGAGGGGGGACGG
GGTAAACAGGGGAAGGAAACAAGAGAACACTGATGAACAGACACAAACCGACCAACGG
GGAAACGGCGCTCGGGCAAGACAAGCGCAGAGCAGAAAAAGAAAACCAGAAACCCAGC
AGCGGCAGGCGAAGCGAAGGACTAAGGGGACAAACCCTCACCAGAAACAAGGGAGCC
AACGCACTGAAACACAAGGGCGAACCTGGCGGTCACAACACACCCAAACAGGGGACAA
GAGCGTGGCACGAAGGCCAGAGGAACATGCAGCAACAGACCACCAGAAGAACAGAGTC
GTAGACAGAACCGAACAACCCCGCCACAGCGACAAGGAACGAAAAGGACAAAAGACCA
CCGCGAGACGTACCCACCATGAAGCCTGGGCGTATGCGCGGCGGGCCCGTCGAAAAC
AAAAAACGCAAATCCACGACACGCCGACGCGGGACGGAGAGAGAAGCCACGACCGAC
GGACAAACAAAGGAACCCGGCCGAGAGAGGAACCCGCACACACGAACACCAGAACAGA
GAGCCGAAAGATCAAAGCAGACAAGCAAACAAGAGACCCAATCAGGAACAGGCCCCGT
CAGCGGGCGACGCAACAGCACCAGGAAGACAGGGGAACCACTCAGATGGCGCACCGA
GGGCAAAACAGGGGAACCGGACAGAAAAAACGGCGCGAGGCGACGAGCCGAGAGACA
GCGACGCCAGCCCCGCGAGCAAAGGCTAATCCCCAGCACGCTACGTGTCGCCGGGGT
GAGGCAGCAAGATCTCGCGAACAACGTGGAACCAAAAAGCCTGAGGGGAGGGACGCCA
AAGCACGAAACACACGCCAAGAGGAAGGCAGCAACCTATCGACCGCAAAAGGAGAAGA
ACCCCACAGGAAAATAAAAGTCAACCAGAACCACCCTGAATGAAAAACGAAAGCACGGT
GCCGTTAACCCAAAACAACGAAGGAGGAGGGACGGAAGGGGAAAACGAAAGGCGCAC
GGAGGAAGCGGAGACAAGAAAAGCAGACGGGGCCGGGTTAGGAATATCCCCTCAGGAA
ACGAGCAGAACAGAAAAGGACAAACGGAGCCAGATCAAGTAAATAACACGGAGCATATC
AATGGAACACGGCAGCAAAAGAAGCAGCCAGAAGCCAAGATACCAGCGCCGGACCCGC
CACGCCAACTACAGGAAACCGCAGACAGAGGAGAGCCAAACCCACAGGAGAGCACAAA
ATGACACAAACGAAGGGCAAGATCGGCAAAACTAACCCGCGCACCACGGTAACGGGCG
CAAGAAGCGCCGAACCGGTTAGGTTATTACTGAGTAGTATTTATTTAAGTATTGTTTGTG
CACTTGCCTGCAGGCCTTTTGAAAAGCAAGCATAAAAGATCTAAACATAAAATCTGTAAG
AATTCCTTGTCGATTCTGGCGTTCTTTATTCCATTTGCTTTCGGTTGCGCGATCGTCCCG
ACTTTTCGCCTGAGTTTTAGGGCCCCTTTTCTTTCTTCGAGTTTCCTTCCTTTTCTCTGCT
CGCTCGTCGGCTTGTGTTTGTTCCCCGCCCAGTCGGCGCGCCACCGTTGAGGTGTTTC
CTGCGTTCCTCTCTGTTTCCTTGTCTTAATCCTTCGTTTGGATCTCCTTGTTGGGTGTTCT
TTGGGTTGGTGCTTGTTGGTGCGTTTTTTGGCTGTGGCTCCAGTTGTGTTTGGCCCGCA
TCCCTCGGTTGGCCTTCGTTTCTGGTCCGTTTCGTCGTTTTGCCGGTTGTCGTAGGTCGT
TGGGTTGGTTCTGTGTCGTGCGAGTTCCTTTCCTCCTATTCTTTTGTGGGTGCTGGCTTG
GGTAGGCGTTTTTTTCCATCGCTTTCGGCGGGTTTCGTGTCTGCCCTCGCGGTTTCATG
ATCGGCTCTCTTCGGCCCTTTCCTCTTCCGGCGGTGGATCCTCCACATGGCTCTGACGT
TGTTGGTGTGGGGGCTGCTCGGCGTCGTTGCTCCCTCGGTCCCTCCGCGGTTTTCGTT
GTGGTAGCTTCTTTCGCCAGACTCTCTTGGTCTGGTGCGGCCCTTGCCTTTTTCCTTGGT

TGCGTTCCCACTCTTCCGGCTTTCGTCTCGGCCGTTTGGGCGACTTTATGCCCCCTATT
GCGTCTTTGTCGGGTCATCCCGGTGGCCTTGTGGCTTGCGGGCTTCCCTTTCGTCGCTC
TCTTCTGGGTATTGGTCGCCCGTTTACTGTTACTTGGGGATGCTTGCCTGTGCCTGGGG
TCCTACTTTCTGGCGCTATTGGGTGCTTTTTCGGTTTTTCACAGGTCATTTTTGTGTCTGA
GTCCCTTGTGGGGTCCTCTGTCCGCGTCCCTTGCTCGTTTCGGTTCCCGCGGTCGTTTG
CCTATCCGATCGGCCTTTCTTTGCGACGTTCTTTTCTCTCTGGGGTGGTGTTGTACCCTG
TCGGCCCCTCGGGCTTTTGTTTTGACGTTGTCAGGTTTTAGTTTGGTGCGCTCGACTCTT
GGGGGGCGTGTGGGTCAGCCGTCGCTTACGCCGTGGTTTTGGAGGGCTCGGTTGTAAT
CCTTCTTGCGGCTTGTACCTTTAGGTCGTGCATCGTTTCATCTCCCTTCCGTTAAGCTGC
ATCGTGCGCGTTGAGCGTTCGGTGTTGGTCCAACTCCCTGATATTGTTTTGGCCTTCTTG
CTCTCTTCTTTCCTTTGGCTGTTTGGTTTCCGTATTGGCGTCCCCTCTCGGCGCCCTCGT
TGTCCGTCTTCTGCGCGTTTGTTTTTCGTTCACTCGGTGATGGCTCATGGGAGTTGATTG
CTCTTCTCCGCCTCCTTCTGGTCTCTCTGTTCGTA

Replication protein expression and purification

Cdt1-Mcm2-7, ORC, Cdc6, DDK, S-CDK, Sld3/7, Sld2, Dpb11, Pol ε, Cdc45, GINS, Mcm10, Ctf4, RPA, Pol α and Topo II were purified as described previously (Yeeles et al., 2015). Topo I, RFC, PCNA, Csm3/Tof1, Mrc1 and Pol δ were expressed in budding yeast (see tables 1-4 for details of strains and strain construction). Cells were grown at 30°C to 2-3 x 10^7 cells per ml in YP + 2% raffinose. Protein expression was induced by addition of galactose to 2% and cell growth was continued for 2 hours at 30°C. Cells were harvested, resuspended in lysis buffer (see specific protein purification methods for details), frozen dropwise in liquid nitrogen and crushed either in a Spex SamplePrep 6775 freezermill, or manually using a pestle and mortar cooled with liquid nitrogen. Cell powder was stored at -80°C. All subsequent protein purification steps were conducted at 4°C.

PCNA purification PCNA was purified following overexpression in both *E. coli* and yeast. No significant differences were observed between replication reactions using the *E. coli* and yeast expressed proteins.

PCNA was purified following overexpression in *E. coli* using a method modified from (Ayyagari et al., 1995). Bl21 (DE3) Rosetta, transformed with pET28a-

PCNA (supplemental table 3) (600 ml culture), were grown at 37°C in LB + 100 μg/ml ampicillin + 34 μg/ml chloramphenicol to an OD_{600} of 0.5. Protein expression was induced by addition of IPTG to 0.5 mM and growth was continued for 2.5 hours. Cells were harvested by centrifugation, washed once in 50 mM Tris-HCl pH 7.2, 10% w/v sucrose and resuspended in 20 ml 50 mM Tris-HCl pH 7.2, 10% w/v sucrose + protease inhibitors (0.3 mM PMSF, 7.5 mM Benzamidine, 0.5 mM AEBSF, 1 mM Leupeptin, 10 μg/ml Pepstatin A and 1 μg/ml Aprotinin (Sigma)). All purification steps were performed at 4°C. Cells were lysed via sonication (40%, 5s on/5s off, total 1 min) and cell debris was cleared by centrifugation (235,000g, 4°C, 30 min). Ammonium sulphate was added to 150 mM followed by addition of polymin P to 0.4% from a 10% stock. Following 10 min stirring insoluble material was removed by centrifugation (27,000g, 4°C, 15 min). To the supernatant 0.23 g/ml solid ammonium sulphate was added slowly and the sample was stirred for 10 min prior to centrifugation (48,000g, 4°C, 15 min). Insoluble material was discarded and 0.24 g/ml solid ammonium sulphate was added to the supernatant followed by 10 min stirring and centrifugation (48,000g, 4°C, 15 min). Precipitated material was resuspended in 3 ml 25 mM Tris-HCl pH 7.2, 10% glycerol, 1 mM EDTA and 100 mM NaCl (buffer P + 100 mM NaCl) and the sample dialysed against the same buffer for 1 hour. Sample conductivity was adjusted to the equivalent of buffer P + 150 mM NaCl by dilution in buffer P and was then applied to a 1 ml HiTrap SP FF column and a 1 ml HiTrap heparin column assembled in tandem. The flow through was collected and applied to a 1 ml DEAE column equilibrated in buffer P + 150 mM NaCl. Proteins were eluted with a 25 ml gradient to buffer P + 600 mM NaCl. PCNA containing fractions were pooled, diluted 2-fold in buffer P and applied to a 1 ml MonoQ column equilibrated in buffer P + 150 mM NaCl. PCNA was eluted with a 30 ml gradient to 600 mM NaCl, peak fractions were pooled, concentrated to ~ 400 μl and applied to an S200 column equilibrated in buffer P + 150 mM NaCl. Fractions containing PCNA were pooled,

frozen in liquid nitrogen and stored at -80°C. Protein concentration was determined using the Bradford assay (Bio-Rad), as was the case for all proteins used in this study.

To purify PCNA following expression in yeast, 50 g cell powder was resuspended in 120 ml buffer P + 400 mM NaCl without EDTA. Cell debris was cleared by centrifugation (235,000g, 4°C, 45 min), $CaCl_2$ was added to 2 mM and the sample was filtered through 0.45 μM syringe filters (Millipore). 1.5 ml Calmodulin Affinity Resin was added and the sample incubated for 90 min. Resin was collected, washed extensively in buffer P + 400 mM NaCl without EDTA + 2 mM $CaCl_2$ and bound proteins were eluted in buffer P + 400 mM NaCl + 2 mM EGTA. The sample was diluted in buffer P until the conductivity was equivalent to buffer P + 150 mM NaCl before separation on a 1 ml monoQ column as described for the *E. coli* expressed protein. Peak fractions were pooled and the conductivity of the sample adjusted to buffer P + 150 mM NaCl. The sample was again separated on a 1 ml monoQ as described above except that the gradient was from 150 mM NaCl to 600 mM NaCl over 30 column volumes. Peak fractions were pooled and processed as described for the protein expressed in *E. coli*.

Topo I purification. Cell powder from a 10L culture of yAE42 was diluted 2:1 in 25 mM Tris-HCl pH 7.2, 10% glycerol, 0.02% NP-40-S, 300 mM NaCl (Buffer T + 300 mM NaCl) + protease inhibitors + 1 mM DTT. Cell debris was cleared by centrifugation (235,000g, 4°C, 45 min), $CaCl_2$ was added to 2 mM and the sample was filtered through 0.45 μM syringe filters (Millipore). To the soluble extract 800 μl Calmodulin Affinity Resin (Agilent Technologies) was added and the sample incubated for 90 min. Resin was collected in a 20 ml disposable column (Bio-Rad), washed with 100 ml Buffer T + 300 mM NaCl + 2 mM $CaCl_2$ + 1 mM DTT and resuspended in 1 ml of the same buffer + 100 μg/ml TEV protease. Following a 3-

hour incubation the eluate was collected and any remaining proteins were eluted with 1 ml Buffer T + 300 mM NaCl + 2 mM $CaCl_2$. The eluates were pooled, dialysed for 3 hours against Buffer T + 300 mM NaCl and passed over an ~ 250 μl TALON column (Clontech) equilibrated in the same buffer. The flow through was collected, concentrated to ~ 500 μl and separated through an S200 column equilibrated in Buffer T + 300 mM NaCl + 1 mM DTT. Peak fractions were pooled and concentrated to ~ 1 mg/ml prior to storage.

RFC purification yAE41 cell powder (40g) was diluted ~ 2:1 in 25 mM HEPES-KOH pH 7.6, 10% glycerol, 1 mM DTT, 400 mM NaCl (Buffer R + 400 mM NaCl) + protease inhibitors. Cell debris was cleared by centrifugation (235,000g, 4°C, 1 hour) and $CaCl_2$ was added to 2 mM together with 1.5 ml Calmodulin Affinity Resin. Following 90 min incubation the resin was collected, washed with 80 ml Buffer R + 400 mM NaCl + 2 mM $CaCl_2$ and bound proteins eluted with Buffer R + 400 mM NaCl + 2 mM EDTA + 2 mM EGTA. The eluate was slowly diluted ~ 2-fold by addition of Buffer R before being applied to a 1 ml MonoS equilibrated in Buffer R + 1 mM EDTA + 200 mM NaCl. Proteins were eluted with a 30 ml gradient to Buffer R + 1 mM EDTA + 1 M NaCl. Peak fractions were pooled, concentrated to ~ 400 μl and separated through an S200 column equilibrated in Buffer R + 1 mM EDTA + 150 mM NaCl. RFC containing fractions were pooled and concentrated to ~ 2 mg/ml.

Pol δ purification Cell powder from yAE34 (60g) was thawed and diluted 2-fold in Buffer T + 400 mM NaCl + 1 mM DTT + protease inhibitors. After clearing the cell debris by centrifugation (235,000g, 4°C, 1 hour) the NaCl concentration of the lysate was reduced to 300 mM by dilution in Buffer T + 1 mM DTT + protease inhibitors. $CaCl_2$ was added to 2 mM and the sample was applied to an 800 μl Calmodulin Affinity Resin column under gravity flow. The column was washed with 100 ml Buffer

T + 1 mM DTT + 300 mM NaCl + 2 mM $CaCl_2$ and 10 ml Buffer T + 1 mM DTT + 200 mM NaCl + 2 mM $CaCl_2$ without NP-40-S. Proteins were eluted in Buffer T + 1 mM DTT + 200 mM NaCl + 2 mM EDTA + 2 mM EGTA without NP-40-S. Peak fractions were pooled and applied to a 1 ml HiTrap Heparin column equilibrated in Buffer T + 1 mM DTT + 200 mM NaCl + 1 mM EDTA without NP-40-S. Pol δ was eluted with a 30 ml gradient to 1 M NaCl. Pooled fractions were concentrated to ~ 500 μl and were separated through an S200 column equilibrated in Buffer T + 1 mM EDTA + 1 mM DTT + 150 mM NaCl. Peak fractions were pooled and concentrated prior to snap freeing in liquid nitrogen and storage at -80°C.

Csm3/Tof1 purification Cell powder form a 6 L culture of yAE48 was thawed and diluted 2:1 in Buffer T + 1 mM DTT + 200 mM NaCl. After clearing the lysate by centrifugation (235,000g, 4°C, 1 hour) $CaCl_2$ was added to 2 mM together with 500 μl Calmodulin Affinity Resin and the sample incubated for 90 min. Resin was collected in a 20 ml column (Bio-Rad) and the column washed with 50 ml Buffer T + 1 mM DTT + 200 mM NaCl + 2 mM $CaCl_2$. Resin was resuspended in 800 μl of the column wash buffer and TEV was added to 100 μg/ml. The sample was incubated on ice for 2 hours. Cleaved proteins were eluted and dialysed against 1L Buffer T + 100 mM NaCl. To remove the His-tagged TEV protease the dialysed sample was applied to an ~ 250 μl TALON column equilibrated in dialysis buffer. The flow through was then concentrated to ~ 400 μl and applied to an S200 column equilibrated in Buffer T + 150 mM NaCl. Peak fractions were pooled and concentrated to ~0.7 mg/ml.

Mrc1 purification Cell powder form a 10 L culture of yJY32 was thawed and diluted 2:1 in Buffer T + 0.5 mM DTT + 1 mM EDTA + 400 mM NaCl (Buffer M + 400 mM NaCl) + protease inhibitors. Insoluble material was cleared by centrifugation (235,000g, 4°C, 1 hour) and 1.5 ml FLAG M2 Affinity gel (Sigma) was added to the

supernatant. The sample was incubated for 90 min before the resin was collected in a 20 ml column and was washed with 40 ml Buffer T + 0.5 mM DTT + 1 mM EDTA + 400 mM NaCl. Resin was then resuspended in 10 ml Buffer T + 0.5 mM DTT + 400 mM NaCl + 10 mM magnesium acetate + 1 mM ATP and the sample incubated for 10 min. Resin was collected and washed with 40 ml Buffer T + 0.5 mM DTT + 1 mM EDTA + 400 mM NaCl. FLAG-tagged proteins were eluted by incubating the resin for 30 min in 1 ml Buffer T + 0.5 mM DTT + 1 mM EDTA + 400 mM NaCl + 0.5 mg/ml 3x FLAG peptide, and a further 10 min in buffer containing 0.25 mg/ml 3x FLAG peptide. Eluates were pooled, slowly diluted ~ 2-fold in Buffer T + 0.5 mM DTT + 1 mM EDTA and applied to a 1 ml MonoQ equilibrated in Buffer T + 0.5 mM DTT + 1 mM EDTA + 200 mM NaCl. Proteins were eluted with a 30 ml gradient to Buffer T + 1 M NaCl. Mrc1 containing fractions were pooled and dialysed for 2.5 hours against Buffer T with 40% glycerol + 0.5 mM DTT + 1 mM EDTA + 150 mM NaCl.

Pol ε – Δcat expression and purification

The codon optimised Pol2 gene (Yeeles et al., 2015) was amplified with primers AJ34 and AJ32 to remove the nucleotide sequence encoding the first 1262 amino acids. The full length Pol2 gene was then replaced with the truncated form and the resulting plasmid was used to generate the expression strain (See supplemental tables 1-4 for details). The protein was purified using a modified version of the previously published protocol for wild type Pol ε (Yeeles et al., 2015), where the heparin column was replaced by a MonoQ column. Following elution from the calmodulin affinity gel the protein was applied to a 1 ml MonoQ equilibrated in 25 mM HEPES-KOH pH 7.6, 10% glycerol, 2 mM 2-mercaptoethanol + 400 mM KOAc. Pol ε – Δcat was eluted with a 20 ml gradient from 400 mM to 1.5 M KOAc in column equilibration buffer. Peak fractions were pooled and separated through an S200 as previously described (Yeeles et al., 2015).

Bead-bound replication reactions

All reaction steps were conducted at 30°C with agitation (1200 rpm). To ensure origin specificity (Yeeles et al., 2015) ORC was pre-bound to DNA in a buffer (35 μl) containing 25 mM HEPES-KOH pH 7.6, 50 mM KCl, 10 mM $Mg(OAc)_2$, 5% Glycerol, 2 mM ATP, 1 mM DTT, 100 μg/ml BSA, 12.5 ng/μl linear DNA beads and 2.5 nM ORC. The supernatant was removed, the beads washed twice with 150 μl 40 mM HEPES-KOH pH 7.6, 10 mM $Mg(OAc)_2$, 5% glycerol, 0.02% NP-40-S and 300 mM KOAc and a new buffer (35 μl) containing 25 mM HEPES-KOH pH 7.6, 100 mM K-glutamate, 10 mM $Mg(OAc)_2$, 0.02% NP-40-S, 5% glycerol, 2 mM DTT, 5 mM ATP, 45 nM Cdc6, 100 nM Cdt1•Mcm2-7 was added and the mix incubated for 20 min. The buffer was removed and replaced with 35 μl 25 mM HEPES-KOH pH 7.6, 100 mM K-glutamate, 10 mM $Mg(OAc)_2$, 0.02% NP-40-S, 5% glycerol, 2 mM DTT, 5 mM ATP and 25 nM DDK. After a 20 min incubation the reaction was divided into three 10 μl aliquots. Replication buffer was added and reactions were initiated by addition of proteins (typically 10-15% of the final reaction volume) to give final reaction volumes of 20 μl containing 25 mM HEPES-KOH pH 7.6, 100 mM K-glutamate, 10 mM $Mg(OAc)_2$, 0.02% NP-40-S, 2.5% glycerol, 2 mM DTT, 100 μg/ml BSA, 3 mM ATP, 200 μM CTP, GTP, UTP, 80 μM dCTP, dGTP, dATP, dTTP, 12.5 nM DDK, 30 nM Dpb11, 200 nM GINS, 40 nM Cdc45, 20 nM Pol ε, 5 nM Mcm10, 20 nM S-CDK, 100 nM RPA, 20 nM Ctf4, 20 nM Pol α, 30 nM Sld3/7 and 45 nM Sld2. Either [α-^{32}P] labelled dCTP, dTTP or dATP (Perkin Elmer) were added to 40 nM to label nascent replication products.

Data analysis

All gels were scanned using a typhoon phosphorimager. Data were processed in ImageJ after converting the .gel files to 16-Bit Tiff files using the Linearize GelData command. For pulse-chase experiments the positions of the peaks were assigned

manually. Maximum product lengths were derived in GraphPad Prism by calculating and then smoothing the first derivative of lane profiles. For each experiment the product front was defined as being located at a specific value of the first derivative (typically 10-20% of the maximum value), at which point product intensity was rapidly increasing. The same first derivative value was used for all time points within an experiment. Data were fit to linear regressions and the slope of the regression was used to calculate replication rates.

Supplemental References

Ayyagari, R., Impellizzeri, K.J., Yoder, B.L., Gary, S.L., and Burgers, P.M. (1995). A mutational analysis of the yeast proliferating cell nuclear antigen indicates distinct roles in DNA replication and DNA repair. Mol Cell Biol *15*, 4420-4429.

Coster, G., Frigola, J., Beuron, F., Morris, E.P., and Diffley, J.F.X. (2014). Origin Licensing Requires ATP Binding and Hydrolysis by the MCM Replicative Helicase. Mol Cell *55*, 666-677.

Frigola, J., Remus, D., Mehanna, A., and Diffley, J.F.X. (2013). ATPase-dependent quality control of DNA replication origin licensing. Nature *495*, 339-343.

Liachko, I., Youngblood, R.A., Keich, U., and Dunham, M.J. (2013). High-resolution mapping, characterization, and optimization of autonomously replicating sequences in yeast. Genome research *23*, 698-704.

Sharp, P.M., and Li, W.H. (1987). The codon Adaptation Index--a measure of directional synonymous codon usage bias, and its potential applications. Nucleic Acids Res *15*, 1281-1295.

Yeeles, J.T., Deegan, T.D., Janska, A., Early, A., and Diffley, J.F.X. (2015). Regulated eukaryotic DNA replication origin firing with purified proteins. Nature *519*, 431-435.
